# Effects of high-intensity interval training and moderate-intensity continuous training on type 2 diabetes mellitus: a meta-analysis and systematic review

**DOI:** 10.3389/fendo.2026.1876685

**Published:** 2026-07-07

**Authors:** Fan Zeng, Mengke Zhu, Yang Yang, Fatao Wang

**Affiliations:** School of Leisure Sports and Tourism, Beijing Sport University, Beijing, China

**Keywords:** high-intensity interval training, lipid metabolism, meta-analysis, moderate-intensity continuous training, type 2 diabetes

## Abstract

This meta-analysis compared the effects of high-intensity interval training (HIIT) and moderate-intensity continuous training (MICT) on type 2 diabetes (T2DM). A systematic search of the PubMed, Web of Science, Cochrane Library and Embase databases (from the inception of each database to 16 November 2025) identified a total of 21 randomized controlled trials involving 792 participants. A pooled analysis was conducted using standardized mean differences (SMD) and 95% confidence intervals. The results showed no significant differences between HIIT and MICT in terms of glycated hemoglobin (HbA1c), fasting blood glucose, HOMA-IR, body mass index (BMI), total cholesterol (TC), triglycerides (TG), low-density lipoprotein cholesterol (LDL) or blood pressure. However, HIIT demonstrated a significant advantage in improving high-density lipoprotein cholesterol (HDL) (SMD 0.44, p = 0.023). Subgroup analyses indicated that moderate training duration (20–30 minutes), lower weekly training frequency (≤3 sessions) and shorter intervention duration (≤8 weeks) optimized the benefits of HIIT on HDL. These findings suggest that HIIT is an effective alternative to MICT for patients with type 2 diabetes, particularly those with time constraints and whose primary metabolic abnormality is characterized by low HDL levels. Personalized exercise prescriptions should consider training duration and weekly training frequency as key modifiers.

**Systematic review registration:**

https://www.crd.york.ac.uk/PROSPERO/, identifier CRD420261343976

## 1 Introduction

Type 2 diabetes (T2DM) has become one of the most pressing global public health challenges of the 21st century. According to the 10th edition of the Diabetes Atlas published by the International Diabetes Federation (IDF), approximately 537 million adults (aged 20–79) worldwide had diabetes in 2021, representing a prevalence of 10.5%; this figure is projected to rise to 783 million by 2045 (1).The pathophysiology of type 2 diabetes is multifactorial, involving progressive HOMA-IR in peripheral tissues (particularly skeletal muscle, liver and adipose tissue), accompanied by dysfunction and failure of pancreatic β-cells. As the disease progresses, patients face a significantly increased risk of developing severe microvascular complications (retinopathy, nephropathy, neuropathy) and macrovascular complications (coronary artery disease, stroke), which substantially increase morbidity and mortality (2). The risk of these complications is closely linked to glycemic control: for every 1% reduction in glycated hemoglobin (HbA1c), the risk of microvascular complications decreases by 37% and diabetes-related mortality by 21% (3, 4). Importantly, parallel improvements in lipid metabolism (e.g., HDL elevation, LDL and TG reduction), insulin sensitivity, and blood pressure exert independent protective effects against diabetic complications, underscoring the necessity of comprehensive metabolic management rather than glycemic control alone (5). This highlights the critical importance of identifying effective, sustainable and time-efficient interventions to optimize comprehensive metabolic control and prevent long-term complications.”

To curb the progression of these complications and improve long-term prognosis, structured exercise training has been established as a cornerstone strategy in the management of type 2 diabetes, forming a complementary intervention system alongside pharmacotherapy. Within these frameworks, moderate-intensity continuous training (MICT) has long occupied a central position in international clinical guidelines due to its robust evidence base and favorable safety profile (6).The American Diabetes Association (ADA) 2016 position statement explicitly recommends that people with diabetes engage in at least 150 minutes of moderate-intensity aerobic exercise per week (7).This training regimen typically involves sustained aerobic exercise for 30 minutes or more at an intensity of 50–75% of maximum oxygen uptake (VO_2_max) or maximum heart rate (8). Randomized controlled trials have demonstrated that MICT effectively reduces fasting blood glucose, HbA1c levels and blood pressure in people with type 2 diabetes, whilst improving insulin sensitivity (4, 9). However, the time-consuming nature of MICT—requiring 45–60 minutes per session, several times a week—presents a significant barrier for those with busy schedules (10).Research has identified a lack of time as the most common barrier to sustained participation in exercise among people with diabetes, whilst physical limitations and insufficient emphasis by healthcare professionals are also significant factors (10). Recent studies confirm that lack of time remains the most common barrier to sustained exercise participation among people with diabetes, with approximately 90% of patients failing to meet physical activity recommendations (11). This adherence challenge is particularly concerning given that sustained glycemic control is essential for complication prevention.

Given these limitations, high-intensity interval training (HIIT) has emerged as a highly promising alternative. HIIT is characterized by repeated bouts of high-intensity exercise (typically at 80% or more of maximum heart rate or 80% or more of maximum oxygen uptake), interspersed with periods of low-intensity recovery or rest (12). Its unique advantage of ‘time for results’—typically requiring only 20 to 30 minutes per session—effectively addresses the primary barriers to exercise adherence, whilst potentially delivering equivalent or even superior metabolic benefits. Early evidence demonstrated that functional HIIT can safely and effectively improve β-cell function in adults with T2DM (13), establishing its potential for preserving endogenous insulin secretion. Recent evidence further confirms the role of HIIT in mitigating diabetes-related complications. Studies indicate that 12 weeks of high-intensity interval training (HIIT) under professional supervision can improve cardiovascular autonomic function, as evidenced by enhanced arterial pressure reflex sensitivity and reduced postural blood pressure changes in overweight men with type 2 diabetes (14). Furthermore, a 2025 systematic review and meta-analysis confirmed that exercise training, including HIIT, significantly influences cardiac autonomic function in patients with type 2 diabetes, particularly by improving the Valsalva ratio and baroreflex sensitivity (15). The systematic review also confirmed that HIIT significantly improves glucose and lipid metabolism in patients with type 2 diabetes, with large effect sizes observed for HbA1c, TC and HDL (16). Home-based high-intensity interval training (HIIT) programs have further demonstrated efficacy in improving cardiorespiratory fitness, showing potential for practical application (17). These multifaceted benefits make HIIT a comprehensive strategy that balances metabolic control with the prevention of complications.

Despite these encouraging advances, evidence comparing the efficacy of high-intensity interval training (HIIT) with moderate-intensity continuous training (MICT) remains mixed. A 2025 umbrella review of 10 systematic reviews concluded that HIIT significantly improves glycated hemoglobin (HbA1c) and cardiorespiratory fitness compared with MICT in type 2 diabetes (3). Similarly, a 2024 meta-analysis of 22 RCTs confirmed that HIIT yields larger effect sizes for improving glucose and lipid metabolism in these patients (16). Conversely, a 2024 RCT in adults with prediabetes reported that, although both modalities improved glycemic control and aerobic capacity, MICT was superior in reducing visceral fat, triglycerides and diastolic blood pressure (18). Furthermore, meta-analyses have observed largely comparable effects of the two modalities on body composition, blood pressure and cardiometabolic risk factors, with neither demonstrating consistent superiority across all parameters (8, 19–21). To date, no comprehensive meta-analysis has systematically compared their effects on the full spectrum of metabolic outcomes—including glycemic control, insulin sensitivity, body composition, lipid profile and cardiovascular parameters—in a single analysis of adults with type 2 diabetes.

Consequently, this meta-analysis aims to systematically compare the effects of HIIT and MICT on blood glucose control, insulin sensitivity, body composition, lipid metabolism and cardiovascular parameters in adults with type 2 diabetes. By synthesizing the evidence from existing randomized controlled trials, this study aims to determine whether HIIT provides superior metabolic benefits compared to conventional MICT, and to identify potential moderators of treatment response, ultimately providing evidence-based guidance for clinicians in the formulation of personalized exercise prescriptions.

## 2 Materials and methods

This systematic review and meta-analysis were conducted in accordance with the Preferred Reporting Items for Systematic Reviews and Meta-Analyses 2020(PRISMA 2020) guidelines (22). The study protocol was preregistered in PROSPERO (registration number:CRD420261343976). We confirm that all analyses were conducted in strict accordance with the pre-registered protocol. No amendments to the protocol were made after registration, and no deviations from the pre-specified eligibility criteria, outcome definitions, or statistical analysis plan occurred during the conduct of this review. As this meta-analysis exclusively used aggregated data extracted from previously published randomized controlled trials, no original human participant data were collected, and therefore ethical approval and informed consent were not required.

### 2.1 Search strategy

We conducted a comprehensive literature search using four major electronic databases: PubMed, Web of Science, the Cochrane Library and Embase. The search covered studies published from the inception of the databases up to 16 November 2025. The search strategies were constructed using a combination of MeSH terms and free-text keywords connected by Boolean operators (AND, OR), tailored to each database’s syntax and indexing system. No additional filters or restrictions (e.g., language, publication year, study type) were applied at the search level; all potentially relevant records retrieved by the search strings were exported for manual screening against the predefined eligibility criteria. Two authors (F.Z. and Y.Y.) independently conducted the literature search and study screening, resolving any discrepancies through discussion with the third author (M.Z.) until consensus was reached. The complete, line-by-line search strategies for all databases are provided in **Supplementary Table 2**. Grey literature and unpublished studies were not searched.

### 2.2 Inclusion and exclusion criteria

The inclusion criteria were defined using the Population, Intervention, Control, Outcome, and Study Design (PICOS) framework: (1) Population: participants diagnosed with type 2 diabetes mellitus (T2DM); (2) Interventions: HIIT, defined as repeated bouts of vigorous to very vigorous exercise (≥80% HRR, ≥80% HRmax/HRpeak, ≥80% VO_2_max or RPE ≥15), interspersed with recovery periods; the structure of work and rest intervals, duration of individual training sessions, weekly training frequency and total duration of the intervention were reported. all characteristics are listed in **Supplementary Table 1**; (3) Control group:MICT, defined as continuous aerobic exercise without structured recovery intervals, with an intensity range of 40%–70% of heart rate reserve (HRR), 50%–70% of maximum oxygen uptake (VO_2_max), 60%–70% of maximum heart rate (HRmax/HRpeak), or 11–15 on the rating of perceived exertion (RPE) scale. (4) Outcome measures: Core metabolic indicators including HbA1c, fasting blood glucose, and HOMA-IR; (5) Study design: Randomized controlled trial (RCT) design.

Exclusion criteria include: (1) literature published in languages other than English. this restriction was applied because the authors lacked the resources for accurate translation and data extraction from non-English texts, potentially introducing language bias; (2) review articles or conference abstracts; (3) studies involving animal models; (4) studies for which the full text is not available; (5) outcome measures that cannot be converted into mean and standard deviation (SD) values.

### 2.3 Data extraction

Two authors (F.Z. and Y.Y.) independently extracted data using a standardized form. For this review, a formal inter-rater agreement statistic, such as Cohen’s kappa, was not calculated; instead, any discrepancies between reviewers were resolved through discussion and consensus with a third author (M.Z.). The data extracted included: (1) study characteristics (first author, year of publication, sample size); (2) details of the intervention (type of intervention, duration, frequency, duration of a single session, weekly duration, and exercise intensity parameters such as %HRmax, %VO_2_max, heart rate reserve, and RPE); (3) participant characteristics (age, body mass index [BMI], and, where reported, diabetes duration, smoking status, comorbidities [cardiovascular disease, hypertension, dyslipidemia], medication use [antidiabetic, antihypertensive, lipid-lowering agents], and cardiorespiratory fitness metrics) to characterize sample heterogeneity and generalizability; (4) outcome measures (changes in values of outcome measures such as HbA1c, fasting blood glucose and HOMA-IR before and after the intervention, along with their within-group statistical significance). The majority of included studies used the original HOMA-IR model, with a minority using the updated HOMA2-IR; for consistency, we refer to this outcome as HOMA-IR throughout the manuscript.

### 2.4 Quality assessment

Risk of bias for the included studies was independently evaluated by two authors (F.Z. and Y.Y.) using the original Cochrane Risk of Bias (RoB) tool for randomized trials (23, 24). Seven domains were assessed: (1) random sequence generation, (2) allocation concealment, (3) blinding of participants and personnel, (4) blinding of outcome assessment, (5) incomplete outcome data, (6) selective reporting, and (7) other bias. Each domain was rated as “low risk”, “high risk”, or “unclear risk” (25). Discrepancies were resolved by discussion with a third author(M.Z.).

GRADE was not applied, as this meta-analysis exclusively synthesized RCTs comparing two active exercise interventions. The Cochrane Risk of Bias tool was deemed sufficient for assessing internal validity, consistent with standard practice in exercise intervention meta-analyses.

### 2.5 Statistical analysis

For each study, we extracted the mean change from baseline and its corresponding standard deviation (SD). Where the standard deviation of the change score was not directly reported, we estimated it using a pre-post correlation coefficient of r = 0.5, combined with the pre- and post-intervention standard deviations, in line with Cochrane’s recommendations for exercise intervention trials. Where only the standard error or 95% confidence interval was reported, we calculated the standard deviation retrospectively.

We used the standardized mean difference (SMD) and its 95% confidence interval as the measure of effect. Although HbA1c is typically reported as a percentage, the units of measurement for other primary outcome measures—including FBG, TG, TC, LDL and HOMA-IR—were not standardized. Given that this meta-analysis combines ten different metabolic outcome measures, the standardized mean difference (SMD) was selected to ensure methodological consistency and to avoid *post-hoc* unit conversion based on the assumption that results from independent laboratories are equivalent.

Heterogeneity was assessed using the I² statistic and the Q-test p-value. Given the anticipated clinical heterogeneity across exercise intervention trials (e.g., variations in HIIT protocols, participant characteristics, baseline fitness levels, and outcome assessment methods), a random-effects model was used for all primary meta-analyses, consistent with recommendations that model selection should be based on the expected distribution of effect sizes rather than on *post-hoc* heterogeneity tests (24, 26). The I² statistic was used to guide the decision to conduct additional exploratory analyses; specifically, subgroup analyses, sensitivity analyses, and funnel plots were performed when I² exceeded 50% to identify potential sources of substantial heterogeneity (24). These subgroup analyses were prespecified and stratified by duration of a single training session, weekly frequency, and intervention duration (27).

Subgroup analyses were stratified by frequency (less than 3 times per week vs. 3 or more times per week), duration of training (less than 20 minutes, 20–30 minutes, and 30 minutes or more), and duration of the program (≤8 weeks vs. >8 weeks). These thresholds were determined *a priori* based on standard exercise prescription categories and previous systematic reviews in this field (21, 28).

Publication bias and small-study effects were assessed using funnel plots, Egger’s linear regression test (29), and sensitivity analyses with the one-by-one exclusion method (26). The trim-and-fill method was not employed due to the limited number of studies and its questionable performance with continuous outcomes in exercise meta-analyses. These assessments were performed for HDL (the sole outcome demonstrating a significant between-group difference) and TC (an outcome exhibiting substantial heterogeneity, I² = 64.2%). These assessments were not conducted for the remaining outcomes, as their consistent null effects and low heterogeneity (I² < 50%) indicated robust estimates unlikely to be altered by publication bias or single-study influence (24). All analyses were performed using Stata 18.0 (StataCorp, College Station, TX, USA). Meta-analyses were conducted using the metan command, funnel plots using metafunnel, Egger’s test using metabias, and sensitivity analysis using metaninf, with the random-effects model (DerSimonian–Laird method) and SMD specification.

## 3 Results

### 3.1 Study selection

**Figure 1:**
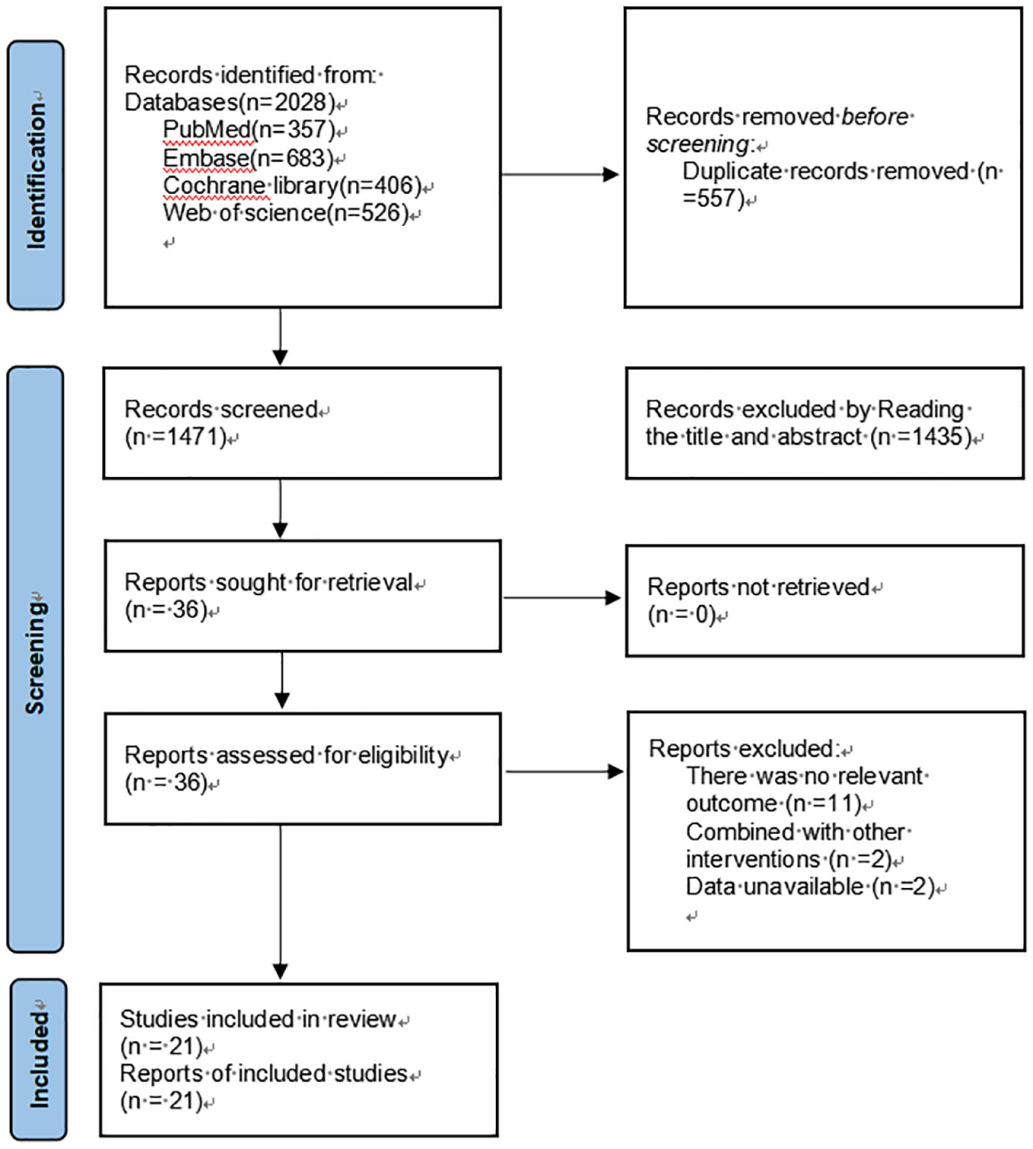
PRISMA flowchart of study selection.

As shown in **Figure 1**, an initial database search identified a total of 2,028 records. After removing duplicate entries, 1,471 articles were retained. Following screening of titles and abstracts, 1,435 studies that did not meet the inclusion criteria were excluded. After a full-text assessment of the remaining 36 studies, 15 were excluded for the following reasons: (1) data unavailable (n=2); (2) lack of relevant outcome measures (n=11); (3) combination with other interventions (n=2). A total of 21 studies were ultimately included (9, 28, 30–48).

### 3.2 Characteristics of the included studies

**Supplementary Table 1** summarizes the key characteristics of the included studies. Five of these studies were conducted in Australia (9, 33, 35, 40, 48), four in Iran (32, 42, 43, 47), Brazil (36, 41, 46) and Canada (28, 30, 31) have three each, Two each for China (44, 45) and Portugal (38, 39);one each in Turkey (37), United States (34).This study included a total of 792 participants diagnosed with type 2 diabetes (T2DM). The study comprised 413 participants in the intervention group and 379 in the control group. The duration of the intervention ranged from 3 to 48 weeks (mean: 13.35 weeks), with a frequency of 1 to 5 sessions per week (mean: 3.1 sessions). One of the studies did not report detailed intervention parameters.

### 3.3 Meta-analysis

This study included a total of 21 randomized controlled trials involving 792 patients with type 2 diabetes and systematically compared the effects of high-intensity interval training (HIIT) and moderate-intensity continuous training (MICT) on nine key metabolic outcome measures. The results of the meta-analysis showed that the two training regimens were comparable in terms of most measures, with a significant between-group difference observed only for HDL.

#### 3.3.1 Glycemic control parameters

HIIT and MICT were equally effective in improving HbA1c (%) (**Supplementary Figure 1**), with no statistically significant difference [SMD, 0.00 (95% CI, −0.19 to 0.19), p = 0.983, I² = 0.0%]. Heterogeneity between studies was extremely low (I² = 0.0%), indicating a high degree of consistency in the results.

There was also no significant difference between HIIT and MICT in terms of their effects on improving FBG [SMD, 0.04 (95% CI, −0.15 to 0.23), p = 0.688, I² = 0.0%]. There was no heterogeneity between studies (I² = 0.0%), and the results are robust and reliable (**Supplementary Figure 2**).

#### 3.3.2 Indicators of insulin sensitivity

HIIT and MICT showed similar effects in improving HOMA-IR (**Supplementary Figure 3**), with no statistically significant difference [SMD, −0.11 (95% CI, −0.43 to 0.21), p = 0.516, I² = 42.8%]. There was low to moderate heterogeneity among the studies, which may be related to differences in baseline HOMA-IR levels, intervention duration, and HIIT protocols across the studies.

#### 3.3.3 Body composition measures

There was no significant difference in the effect of the two training modes on BMI [SMD, 0.08 (95% CI, −0.14 to 0.31), p = 0.477, I² = 26.1%]. There was moderate heterogeneity between studies, which may be attributed to differences in baseline BMI levels, dietary control, and intervention duration across studies (**Supplementary Figure 4**).

#### 3.3.4 Lipid metabolism parameters

HIIT and MICT were equally effective in reducing TC, with no statistically significant difference [SMD, −0.22 (95% CI, −0.55 to 0.11), p = 0.188, I² = 64.2%]. There was significant heterogeneity between studies, suggesting considerable variation in the degree of TC improvement across different studies, which may be related to baseline lipid levels, statin use and the intensity of dietary intervention (**Supplementary Figure 5**). To explore sources of this heterogeneity, we performed subgroup analyses stratified by session duration, weekly frequency, and intervention duration (**Supplementary Figures 6–8**). The results indicated that short-duration HIIT (≤20 min per session) was associated with a significant reduction in TC compared with MICT [SMD, −0.58 (95% CI, −1.04 to −0.13)], whereas moderate-duration (20–30 min) [SMD, 0.04 (95% CI, −0.30 to 0.39)] and long-duration (>30 min) [SMD, −0.05 (95% CI, −0.53 to 0.43)] sessions showed no significant between-group differences (**Supplementary Figure 6**). Neither weekly training frequency nor intervention duration significantly moderated the TC response (**Supplementary Figures 7**, **8**). Detailed publication bias and sensitivity analyses for TC are reported in Sections 3.6 and 3.7.

There was no significant difference in the effect of the two training modes on reducing TG levels [SMD, −0.22 (95% CI, −0.49 to 0.05), p = 0.103, I² = 46.1%]. There was moderate heterogeneity between studies (**Supplementary Figure 9**).

HIIT and MICT showed similar effects in improving LDL levels, with no statistically significant difference [SMD, −0.04 (95% CI, −0.28 to 0.21), p = 0.764, I² = 36.8%]. Heterogeneity between studies was low (**Supplementary Figure 10**).

**Figure 2:**
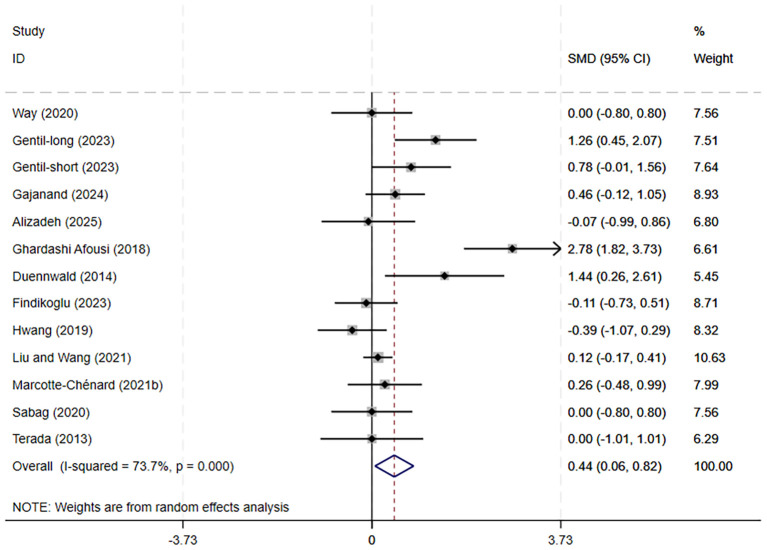
Meta-analysis results of the effects of HIIT and MICT on HDL in patients with type 2 diabetes (9, 28, 30, 32–34, 37, 40–42, 44, 48).

As shown in **Figure 2**, HIIT demonstrated a significantly greater improvement in the outcome measure of HDL [SMD, 0.44 (95% CI, 0.06 to 0.82), p = 0.023, I² = 73.7%] compared with MICT. There was significant heterogeneity between studies (I² = 73.7%), but key moderating factors were identified through subgroup analysis (see Section 3.4 for details). HDL, known as ‘good cholesterol’, exerts multiple cardioprotective effects, including reverse cholesterol transport, anti-inflammatory and antioxidant actions, and improvement of vascular endothelial function. The significant advantage of HIIT on HDL may stem from the more intense metabolic stress induced by its high-intensity nature, which promotes hepatic apolipoprotein A-I synthesis and HDL particle maturation. This finding provides important evidence-based support for the selection of exercise regimens for patients with type 2 diabetes and dyslipidemia.

#### 3.3.5 Cardiovascular parameters

There was no significant difference in the effect of the two training modes on reducing systolic blood pressure [SMD, −0.11 (95% CI, −0.34 to 0.13), p = 0.386, I² = 48.8%]. There was significant heterogeneity between studies, which may be related to differences in baseline blood pressure levels, the use of antihypertensive medication, and the timing of measurements across studies (**Supplementary Figure 11**).

HIIT and MICT were equally effective in improving diastolic blood pressure, with no statistically significant difference [SMD, −0.11 (95% CI, −0.30 to 0.08), p = 0.256, I² = 20.0%]. Heterogeneity between studies was low (**Supplementary Figure 12**).

### 3.4 Subgroup analysis

To investigate the sources of heterogeneity in the effects of HIIT and MICT on HDL levels in patients with type 2 diabetes, this study conducted a pre-specified subgroup analysis based on three dimensions: single-session duration, weekly training frequency, and intervention duration. A random-effects model was used to pool the effects, with the results as follows:

#### 3.4.1 Grouped by session duration

**Figure 3:**
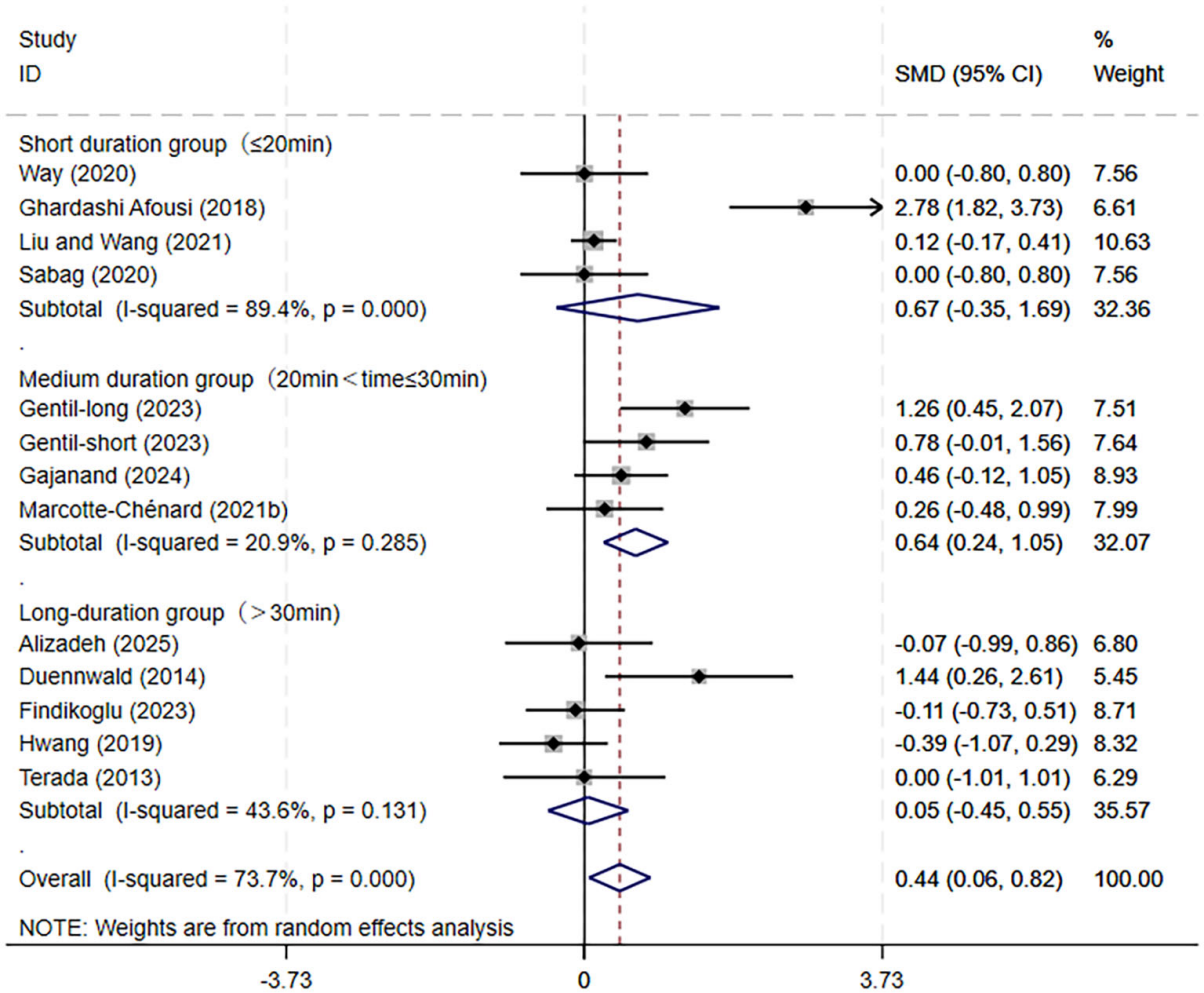
Meta-analysis results on the effect of training duration on HDL in patients with type 2 diabetes (9, 28, 30, 32–34, 37, 40–42, 44, 48).

The included studies were divided into three subgroups based on the duration of a single HIIT session: the short-duration group (≤20 min), the moderate-duration group (20 min < t ≤ 30 min), and the long-duration group (>30 min). These categories correspond to the low-volume, standard, and extended exercise bout classifications commonly applied in HIIT intervention trials and previous systematic reviews in this field (12). As shown in **Figure 3**.

(1) Short-duration group (≤20 min): A total of four studies were included, with a pooled [SMD, 0.67 (95% CI, −0.35 to 1.69), I² = 89.4%, P =0.198]. This subgroup exhibited extremely high heterogeneity, with the 95% CI crossing the line of no effect, indicating that there was no statistically significant difference in the effect of short-duration HIIT versus MICT on improving HDL.

(2) Medium-duration group (20 min < t ≤ 30 min): A total of 4 studies were included, with a pooled [SMD, 0.64 (95% CI, 0.24 to 1.05), I² = 20.9%, P = 0.002]. Heterogeneity between studies was low, and the pooled effect size was statistically significant (P < 0.05), suggesting that moderate-duration HIIT significantly outperformed MICT in terms of its effect on raising HDL.

(3) Long-duration group (>30 min): A total of 4 studies were included, with a pooled [SMD, 0.05 (95% CI, −0.45 to 0.55), I²=43.6%, P = 0.853]. There was moderate heterogeneity between studies, and the pooled effect was not statistically significant; the two training methods were equally effective.

#### 3.4.2 Grouped by weekly training frequency

**Figure 4:**
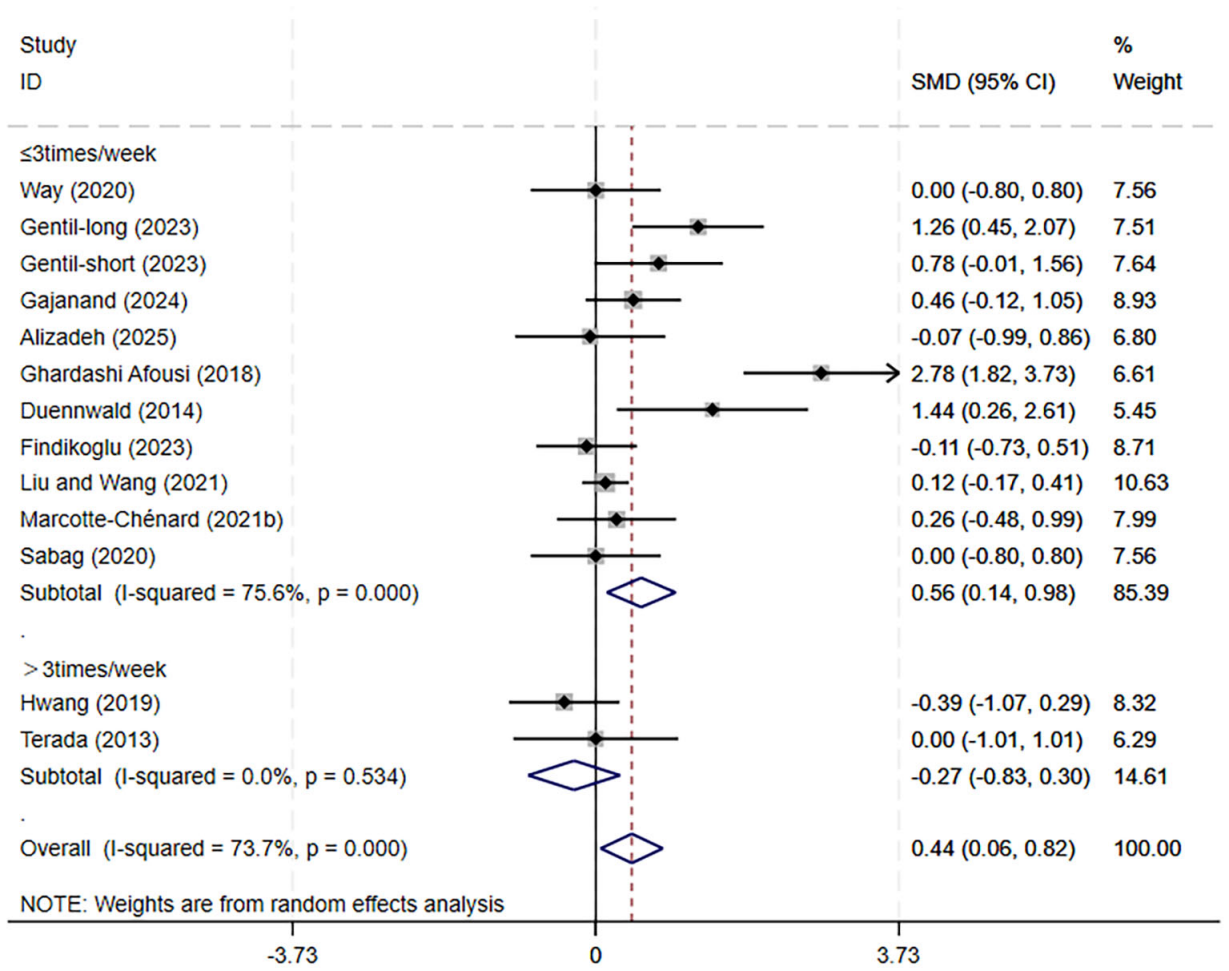
Meta-analysis results on the effect of training frequency on HDL in patients with type 2 diabetes (9, 28, 30, 32–34, 37, 40–42, 44, 48).

The included studies were divided into two subgroups based on weekly training frequency: ≤3 times per week and >3 times per week. This dichotomy was adopted from previous HIIT meta-analyses (21). As shown in **Figure 4**.

(1) ≤3 times per week group: A total of 11 studies were included, with a pooled [SMD, 0.56 (95% CI, 0.14 to 0.98), I²=75.6%, P=0.009]. There was high heterogeneity between studies, and the pooled effect size was statistically significant (P < 0.05), indicating that HIIT performed ≤3 times per week significantly outperformed MICT in terms of its effect on increasing HDL.

(2) >3 sessions/week group: A total of 2 studies were included, with a pooled [SMD, -0.27 (95% CI, -0.83 to 0.30), I²=0.0%, P = 0.354]. There was no heterogeneity between studies, and the pooled effect was not statistically significant; the two training methods were equally effective.

#### 3.4.3 Stratification by intervention cycle

**Figure 5:**
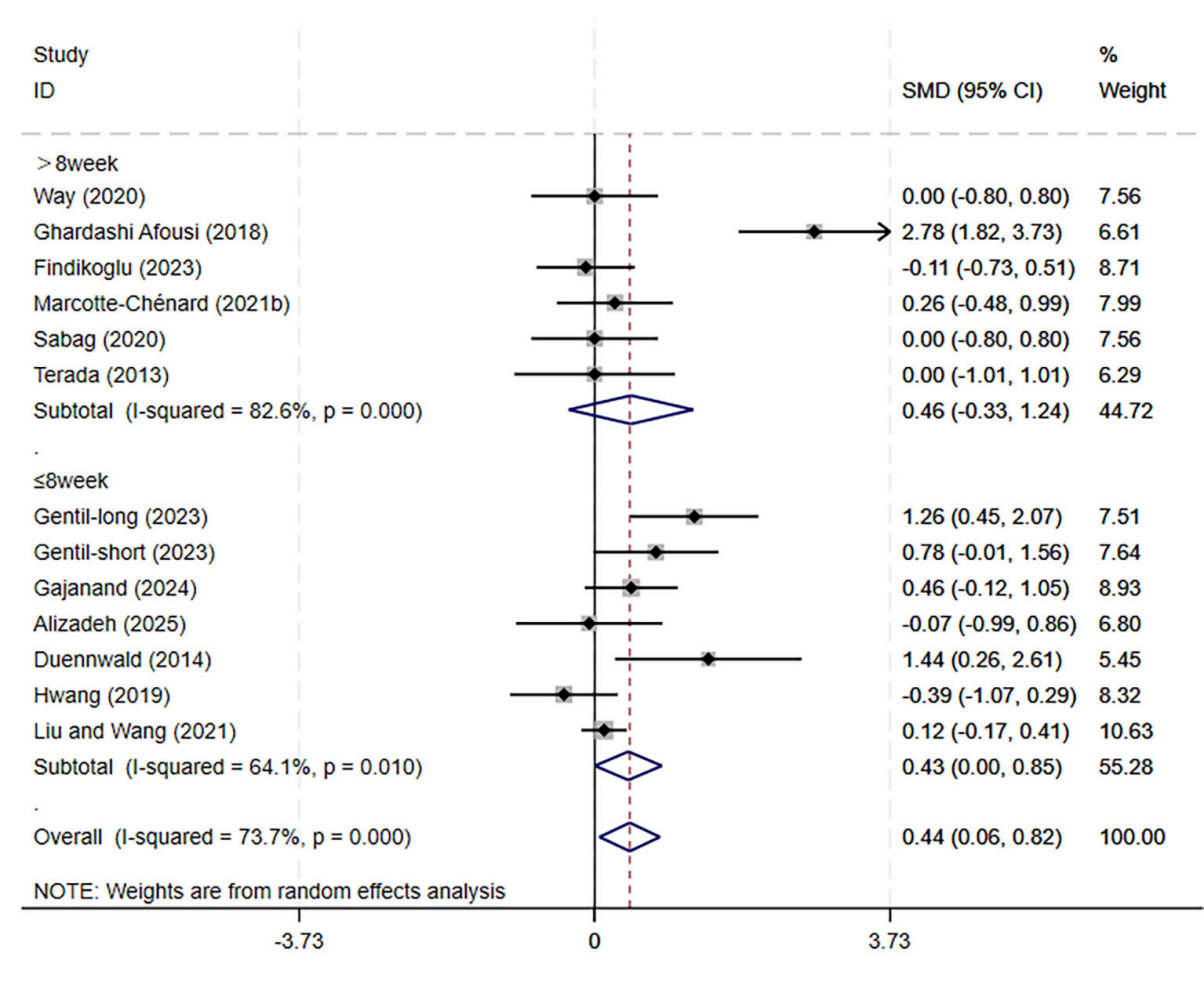
Meta-analysis results on the effect of intervention duration on HDL in patients with type 2 diabetes (9, 28, 30, 32–34, 37, 40–42, 44, 48).

The included studies were divided into two subgroups based on the duration of the intervention: the >8-week group and the ≤8-week group. The 8-week threshold represents the minimum duration typically required to observe initial metabolic adaptations in exercise trials for type 2 diabetes (28). As shown in **Figure 5**.

(1) >8-week group: A total of 6 studies were included, with a pooled [SMD, 0.46 (95% CI: −0.33 to 1.24), I² = 82.6%, P = 0.252]. Heterogeneity between studies was extremely high, and the pooled effect was not statistically significant; the two training methods were equally effective.

(2) ≤8-week group: A total of 7 studies were included, yielding a pooled [SMD, 0.43 (95% CI: 0.00–0.85), I² = 64.1%, P = 0.048]. There was high heterogeneity between studies, and the pooled effect size was statistically significant (P < 0.05), suggesting that short-term (≤8 weeks) HIIT significantly outperformed MICT in improving HDL levels.

### 3.5 Risk of bias

As shown in **Supplementary Figures 13**, **14**, the quality of the included studies was categorized into three levels—high, moderate and low—based on the criteria. Specifically: (1) trials with a high risk of bias in the randomization or allocation concealment processes were rated as low quality; (2) trials with a low risk of bias in both randomization and allocation concealment, and where all other dimensions were rated as low quality or unclear, were considered high quality; (3) all other trials were rated as moderate quality. Of the 21 studies, four were rated as high-quality studies (38–40, 45), Seventeen studies were rated as moderate-quality studies (9, 28, 30–37, 41–44, 46–48).

### 3.6 Publication bias

The funnel plot for HDL outcomes showed no significant asymmetry (**Supplementary Figure 15**). The Egger test confirmed that there was no significant publication bias in the studies on HDL (**Supplementary Table 3**). In contrast, the funnel plot for TC showed significant asymmetry (**Supplementary Figure 17**; Egger’s test, p = 0.017, **Supplementary Table 4**), suggesting potential publication bias. No publication bias assessment (funnel plot or Egger’s test) was performed for the remaining outcomes (HbA1c, FBG, HOMA-IR, BMI, TG, LDL, SBP, and DBP), as these consistently demonstrated null between-group effects and low-to-moderate heterogeneity (I² < 50%), and the presence or absence of publication bias would not change their non-significant interpretation.

### 3.7 Sensitivity analysis

Sensitivity analyses were conducted using the ‘one-by-one’ exclusion method to verify the robustness of the results. For HDL, the primary significant outcome, the results showed (as illustrated in **Supplementary Figure 16**) that, following the exclusion of any single included study, the direction of the 95% CI for the pooled SMD did not change, and the range of variation in the effect size was small. This indicates that the combined effect of the two training regimens on the HDL outcome remains robust and reliable and is not unduly influenced by any individual study. For TC, which exhibited substantial heterogeneity (I² = 64.2%) despite a non-significant between-group effect, sensitivity analysis confirmed that the pooled SMD remained stable and the 95% CI consistently crossed the null line following the exclusion of any single study (**Supplementary Figure 18**), indicating that the null finding was not driven by an individual trial. Sensitivity analyses were not performed for the remaining outcomes (HbA1c, FBG, HOMA-IR, BMI, TG, LDL, SBP, and DBP), as their low-to-moderate heterogeneity (I² < 50%) indicated robust pooled estimates unlikely to be materially altered by the exclusion of individual trials (26).

## 4 Discussion

### 4.1 Summary of key findings

This meta-analysis included 21 randomized controlled trials involving a total of 792 patients with type 2 diabetes and systematically compared the metabolic effects of high-intensity interval training (HIIT) and moderate-intensity continuous training (MICT). Key findings indicate that there were no statistically significant differences between the two training modalities across nine parameters, including HbA1c (SMD = 0.00), FBG (SMD = 0.04), HOMA-IR (SMD = −0.11), BMI (SMD = 0.08), TC (SMD = −0.22), TG (SMD = −0.22), LDL (SMD = −0.04), SBP (SMD = −0.11) and DBP (SMD = −0.11). The sole exception was HDL, where HIIT demonstrated a significantly greater improvement compared with MICT (SMD = 0.44, 95% CI: 0.06–0.82, P = 0.023). Subgroup analyses further revealed that moderate session duration (20–30 minutes), low weekly frequency (≤3 sessions) and short intervention duration (≤8 weeks) optimized the beneficial effects of HIIT on HDL. Overall, HIIT and MICT were comparable in terms of comprehensive metabolic management in patients with type 2 diabetes, but HIIT demonstrated a specific advantage in improving HDL and was more time-efficient.

It is worth noting that although the overall pooled effect of TC did not reach statistical significance (P = 0.188, I² = 64.2%), subgroup analyses stratified by training duration revealed a clear dose-response pattern: compared with MICT, short-duration HIIT (≤20 minutes per session) significantly reduced TC levels [SMD, −0.58 (95% CI,−1.04 to −0.13), p = 0.012, I² = 54.6%], whereas no significant between-group differences were observed for moderate-duration (20–30 minutes) [SMD, 0.04 (95% CI,−0.30 to 0.39), p = 0.807, I² = 0.00%] and long duration (>30 minutes) [SMD, −0.05 (95% CI,−0.53 to 0.43), p = 0.837, I² = 23.3%] training sessions did not show significant differences between groups. Neither the frequency of training per week nor the duration of the intervention significantly modulated the response of TC.

### 4.2 An in-depth analysis of the research findings

HIIT was significantly more effective than MICT in improving HDL levels (SMD = 0.44), a finding closely linked to its specific effects on reverse cholesterol transport (RCT). High-intensity exercise upregulates hepatic apolipoprotein A-I (Apo-AI) synthesis and ABCA1-mediated cholesterol efflux—the initial step in RCT. Exercise training significantly increases HDL-specific phospholipid efflux (HDL-SPE), and this change is strongly correlated with Apo-AI concentration and the number of medium-sized HDL particles, confirming that exercise enhances HDL function via an Apo-AI-dependent mechanism (49). Furthermore, short-term HIIT improves HDL antioxidant capacity (via superoxide dismutase-1 activity) and reduces HDL triglyceride content in patients with T2DM; it maintains HDL function even when plasma Apo-AI levels remain unchanged, suggesting that HIIT enhances HDL quality by optimizing HDL particle composition and functional characteristics (50). Subgroup analyses revealed that moderate single-session duration (20–30 min), low frequency (≤3 sessions) and short intervention duration (≤8 weeks) yielded the best results, exhibiting an inverted U-shaped dose-response relationship: acute metabolic stress activates the Nrf2/PPARα pathway to promote HDL synthesis, whereas excessive exercise induces oxidative stress that inhibits Apo-AI expression (42). Conversely, short-duration HIIT (≤20 min) significantly reduced TC (SMD = −0.58), whereas longer durations did not produce this effect, suggesting that HDL and TC are regulated by independent pathways. Specifically, short-duration high-intensity exercise activates hepatic AMPK, phosphorylating and inhibiting the rate-limiting enzyme of cholesterol synthesis, HMG-CoA reductase (HMGCR), whilst simultaneously suppressing SREBP-2-mediated cholesterol synthesis (51); when exercise duration exceeds 20 minutes, metabolic adaptation shifts towards sustained oxidative metabolism, which may upregulate SREBP-2 and offset the acute cholesterol-lowering effect. This divergent dose-response relationship between HDL and TC supports a mixed prescription strategy—short-duration exercise for lowering TC and moderate-duration exercise for raising HDL.

No significant differences were observed between the two groups for HbA1c, FBG, HOMA-IR, BMI, SBP and DBP; this can be explained by convergent molecular mechanisms: both HIIT and MICT activate the skeletal muscle AMPK–GLUT4 axis, promoting insulin-independent glucose uptake (52). In patients with T2DM, where insulin signaling is impaired, exercise-mediated activation of AMPK and CaMKII promotes GLUT4 translocation to the plasma membrane via TBC1D1/TBC1D4, thereby circumventing the defect in insulin signaling (52). Long-term training also remodels muscle via PGC-1α-mediated mitochondrial biogenesis, producing comparable insulin-sensitizing effects regardless of the intensity regimen (51).

This study makes three significant contributions compared with previous literature. Firstly, within the framework of a direct comparison between HIIT and MICT, this study systematically reveals for the first time that moderate session duration (20–30 minutes), low frequency (≤3 sessions) and a short intervention period (≤8 weeks) are the optimal conditions for HIIT to increase HDL, clearly demonstrating an inverted U-shaped dose-response relationship. Secondly, this study found divergent dose-response relationships between HDL and TC: HDL responds to the Apo-AI/ABCA1 upregulation mechanism induced by moderate-duration HIIT, whereas TC is sensitive only to the AMPK-HMGCR inhibition pathway triggered by short-duration HIIT (≤20 min). This provides the first evidence that different lipid parameters require differentiated exercise strategies. Thirdly, this study strictly limited its analysis to direct head-to-head RCTs in adults with T2DM and employed SMD for effect size pooling. Compared with previous reviews covering broader populations, it provides a comparative benchmark with higher internal validity, laying a methodological foundation for the clinical translation of precision exercise prescriptions.

### 4.3 Clinical and translational significance

Based on the findings of this study, we propose the following tiered exercise prescription strategy: for patients with type 2 diabetes and dyslipidemia (particularly those with low HDL), we recommend a high-intensity interval training (HIIT) program of moderate duration (20–30 minutes), undertaken 2–3 times per week for 8 weeks, which is expected to result in a significant improvement in HDL levels. This program is particularly suitable for patients with time constraints who are unable to complete 150 minutes of moderate-intensity continuous training (MICT) per week. As cardiovascular risk decreases by 2–3% for every 1 mg/dL increase in HDL (53), the improvement in HDL achieved through HIIT has clear clinical value. For patients whose primary lipid abnormality is elevated TC rather than low HDL, short-duration HIIT (≤20 minutes) may offer a specific advantage in reducing total cholesterol and should be considered as a complementary approach. For patients whose primary goal is glycemic control, either training modality may be selected; however, the time-efficiency advantage of HIIT is more pronounced—a cumulative 78 minutes of HIIT per week can achieve glycemic control effects similar to 210 minutes of MICT per week, with better adherence. For elderly patients or those with cardiovascular complications, it is recommended to start with low-volume HIIT (e.g., 10 minutes per session), gradually increasing to moderate duration, whilst closely monitoring cardiovascular responses.

Furthermore, given HIIT’s rapid response characteristics and the potential for a plateau in adaptation, a cyclical training program is recommended: an initial 8-week intensive HIIT intervention to rapidly increase HDL, followed by a maintenance phase alternating between HIIT and MICT, or a cyclical arrangement incorporating resistance training, to delay the onset of the adaptation plateau and sustain long-term metabolic benefits.

### 4.4 Strengths and limitations of the study

The strengths of this study include: strict adherence to the PRISMA guidelines and pre-registration on the Prospero platform, ensuring methodological transparency; the inclusion of 21 RCTs, providing an adequate sample size (792 patients); the use of the standardized Cochrane risk of bias assessment tool, indicating a high overall study quality (4 high-quality studies and 17 moderate-quality studies); the robustness of the results was validated through comprehensive subgroup and sensitivity analyses; and, for the first time, key moderators of the effect of HIIT on HDL (session duration and weekly frequency) were systematically identified, providing evidence-based grounds for the formulation of personalized exercise prescriptions.

The limitations of this study include: Firstly, the follow-up period was insufficient; the included studies primarily involved short-term interventions (averaging 13.35 weeks), and there was a lack of long-term follow-up data, making it impossible to assess the impact of HIIT on hard endpoints (cardiovascular events, mortality) or changes in long-term adherence. Secondly, dietary control was not standardized; most studies did not record dietary interventions in detail, and the interaction between exercise and diet remains unclear, which may have confounded the results of some metabolic indicators. Thirdly, there was heterogeneity in HIIT protocols. Significant differences were observed across studies in work-to-rest ratios, intensity thresholds (ranging from 80% to 95% of maximum heart rate) and exercise types (walking, cycling, functional training). Although these factors were partially controlled for using random-effects models and subgroup analyses, they may still have contributed to the observed heterogeneity. Similarly, the included studies used different versions of the homeostasis model assessment (HOMA-IR and HOMA2-IR) to quantify insulin resistance. Although these versions are highly correlated, subtle algorithmic differences may have contributed to the observed heterogeneity in this outcome. Fourthly, individual differences were not fully explored. There are significant individual variations in how patients with type 2 diabetes respond to exercise, and some patients may exhibit ‘exercise resistance’, the mechanisms of which involve genetic polymorphisms, baseline fitness levels and medication use. This study did not conduct a meta-analysis of individual patient data to explore these differences in depth. Fifthly, the included studies exhibited considerable heterogeneity in baseline patient characteristics, including diabetes duration, cardiorespiratory fitness, comorbidity burden (cardiovascular disease, dyslipidemia, hypertension), and medication regimens. As all included studies were randomized controlled trials, within-study randomization should theoretically balance these factors between HIIT and MICT arms, minimizing confounding at the individual trial level. However, between-study differences in baseline health status and disease severity may limit the generalizability of our findings to specific subpopulations, such as patients with advanced diabetic complications, very low cardiorespiratory fitness, or complex polypharmacy. We did not conduct additional subgroup analyses by these variables because (1) randomization already addresses confounding within each RCT, and (2) these variables were not consistently reported across studies, precluding systematic stratification. Future individual participant data (IPD) meta-analyses could formally examine whether these baseline characteristics modify the relative efficacy of HIIT versus MICT (54). Sixthly, the assessment of publication bias and the evaluation of result robustness were incomplete. Funnel plots, Egger’s regression tests, and sensitivity analyses were performed only for HDL (the sole outcome showing a significant between-group difference) and TC (an outcome with substantial heterogeneity). No publication bias assessment or sensitivity analysis was conducted for the other eight outcomes (HbA1c, FBG, HOMA-IR, BMI, TG, LDL, SBP, and DBP). Consequently, we cannot formally exclude publication bias or rule out the influence of individual studies for these secondary outcomes. However, their consistent null effects and low-to-moderate heterogeneity suggest that the overall conclusions are unlikely to be materially affected. Seventhly, grey literature and unpublished studies were not searched, and this review was restricted to peer-reviewed published articles. While this approach ensured higher methodological quality and more complete data reporting among included studies, it may have introduced publication bias by excluding studies with null or negative results that were not formally published in academic journals. Future updates of this review may benefit from incorporating trial registry data and conference abstracts to further reduce this source of bias. Finally, the review was restricted to English-language publications. Relevant studies published in other languages may have been missed, potentially introducing language bias and limiting the global generalizability of the findings. However, previous empirical methodological studies suggest that language restrictions in conventional medical meta-analyses rarely alter overall conclusions (55).

Future research directions: There is an urgent need to conduct large-scale, multicenter RCTs lasting 2–5 years to assess the impact of HIIT on the progression of diabetic complications and cardiovascular hard endpoints; to develop personalized intervention strategies to overcome exercise resistance; to explore the optimal ratio of HIIT combined with resistance training to achieve synergistic improvements in metabolic and muscular function; and to establish a patient stratification model based on baseline HDL levels to accurately identify those who stand to benefit most from HIIT.

## 5 Conclusions

This meta-analysis of 21 RCTs (792 patients) demonstrated that HIIT was significantly superior to MICT in improving HDL (SMD = 0.44, 95% CI: 0.06–0.82, P = 0.023), with moderate session duration (20–30 min), low weekly frequency (≤3 sessions), and short intervention duration (≤8 weeks) identified as optimal parameters. Although no overall between-group difference was observed for TC, subgroup analyses revealed a divergent dose-response pattern: short-duration HIIT (≤20 min) significantly reduced TC (SMD = −0.58, 95% CI: −1.04 to −0.13), whereas moderate- and long-duration sessions did not. For all other metabolic outcomes—including HbA1c, fasting glucose, HOMA-IR, BMI, TG, LDL, and blood pressure—the two modalities produced comparable effects. In summary, HIIT represents an effective, time-efficient alternative to MICT for patients with type 2 diabetes, particularly those with low HDL or elevated TC as primary metabolic targets. We recommend a tiered prescription: moderate-duration HIIT (20–30 min, 2–3 times/week for ≤8 weeks) for HDL improvement, and short-duration HIIT (≤20 min) for TC reduction. For glycemic control, either modality may be selected based on patient preference and adherence capacity.

## Data Availability

The original contributions presented in the study are included in the article/**Supplementary Material**. Further inquiries can be directed to the corresponding author.
