# Supplementary material for: Effects of high-intensity interval training and moderate-intensity continuous training on type 2 diabetes mellitus: a meta-analysis and systematic review
Source: Front Endocrinol (Lausanne). 2026 Jul 7;17:1876685. doi: 10.3389/fendo.2026.1876685 (PMC13385684; doi:10.3389/fendo.2026.1876685)
Supplement: Supplementary file 1 [file DataSheet1.docx]

Supplementary Material

# Supplementary Figures and Tables

For more information on Supplementary Material and for details on the different file types accepted, please see [here](https://www.frontiersin.org/guidelines/author-guidelines#supplementary-material).

## Supplementary Figures


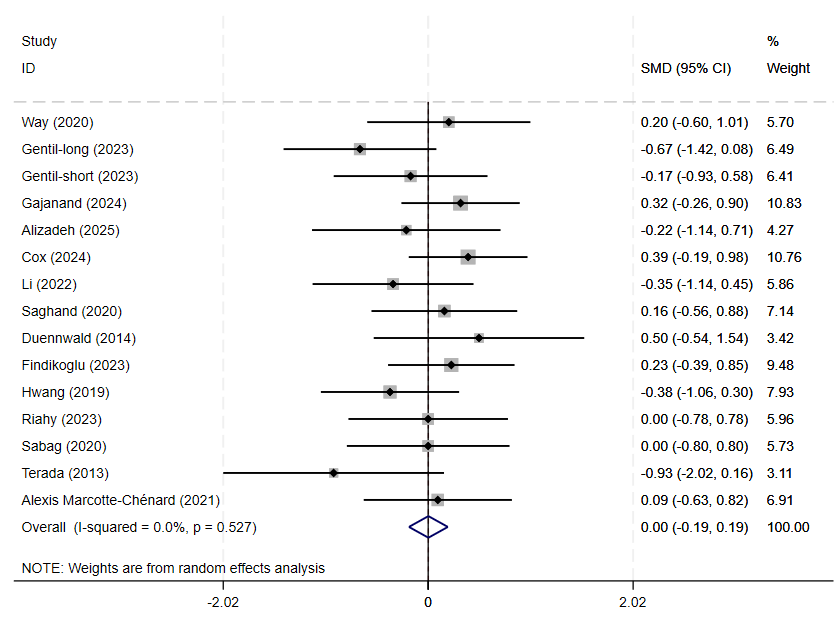


**Supplementary Figure 1.** Meta-analysis of the effects of HIIT and MICT on glycated hemoglobin in patients with type 2 diabetes.


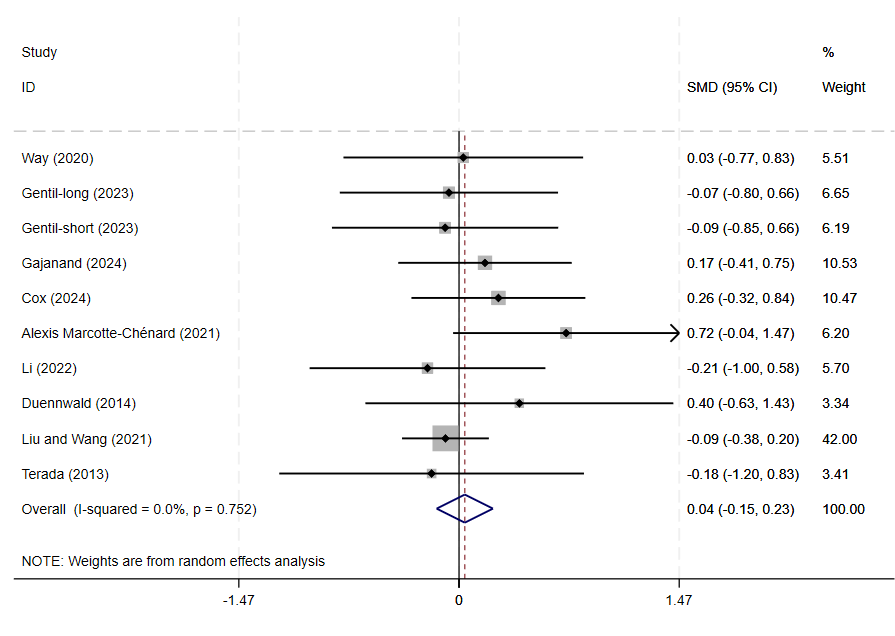


**Supplementary Figure 2.** Meta-analysis of the effects of HIIT and MICT on fasting blood glucose in patients with type 2 diabetes.


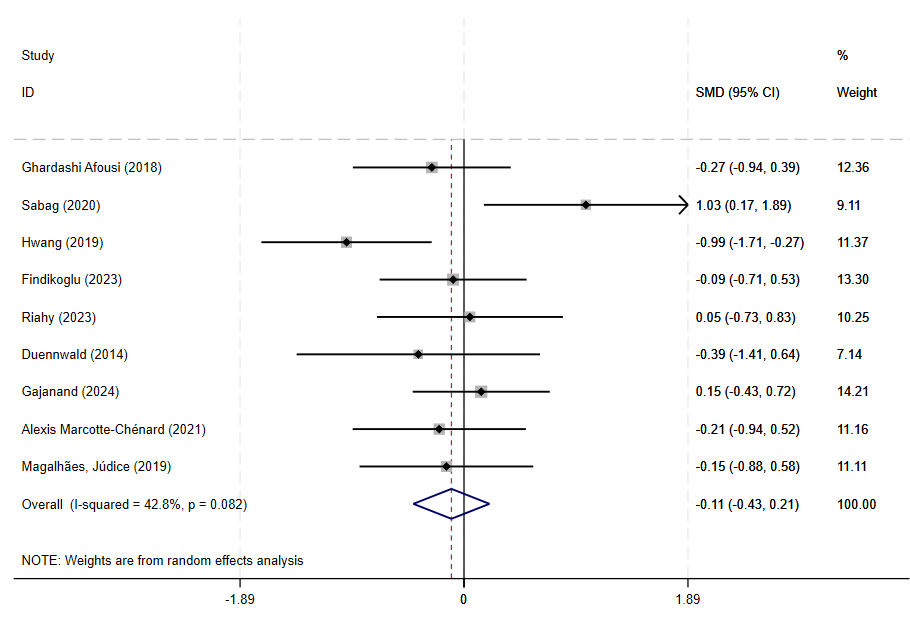


**Supplementary Figure 3.** Meta-analysis of the effects of HIIT and MICT on HOMA-IR in patients with type 2 diabetes.


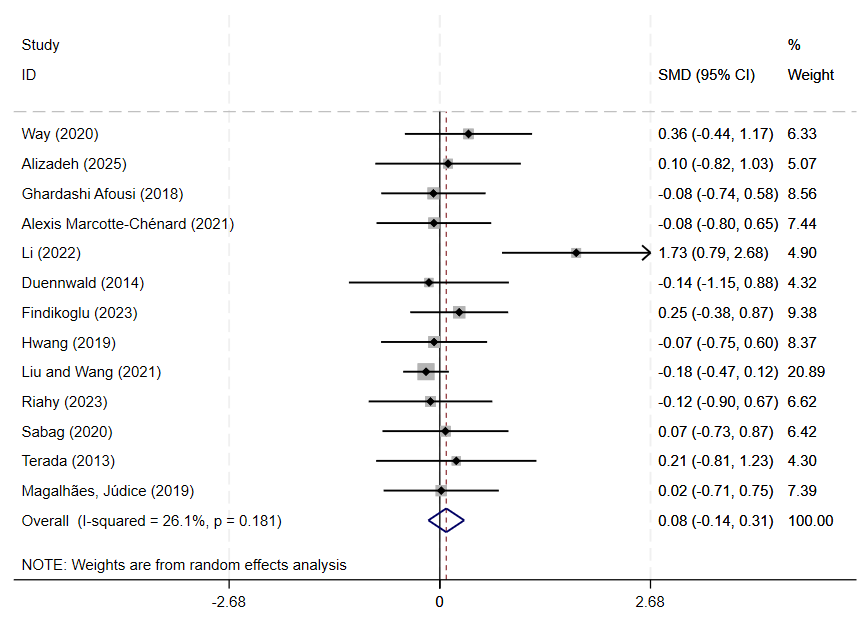


**Supplementary Figure 4.** Meta-analysis of the effects of HIIT and MICT on BMI in patients with type 2 diabetes.


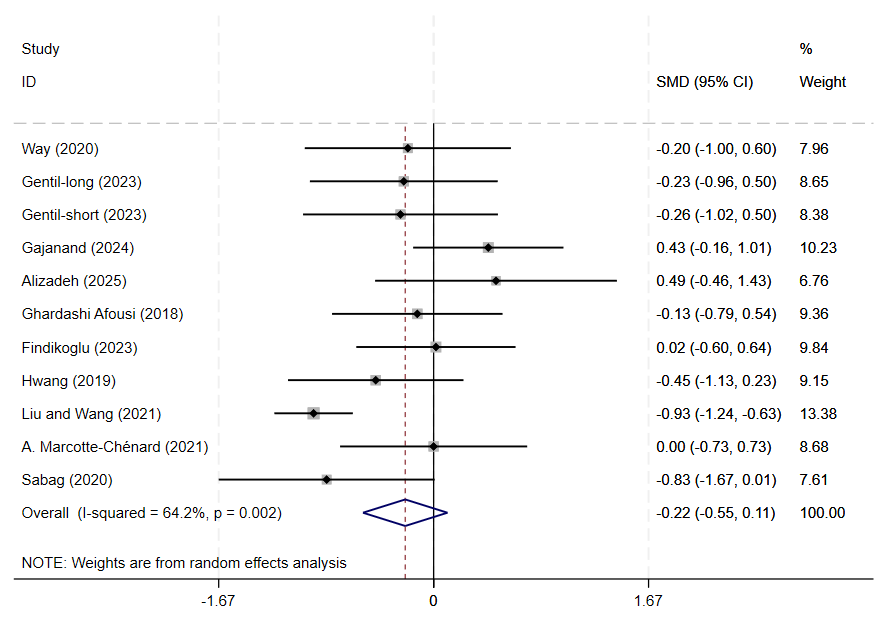
 **Supplementary Figure 5.** Meta-analysis of the effects of HIIT and MICT on TC in patients with type 2 diabetes


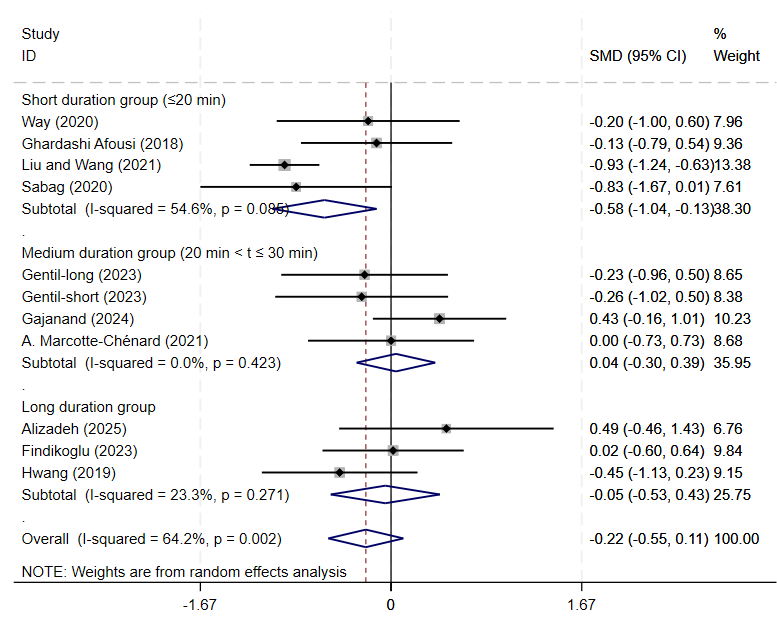


**Supplementary Figure 9.**Meta-analysis results on the effect of training duration on TC in patients with type 2 diabetes


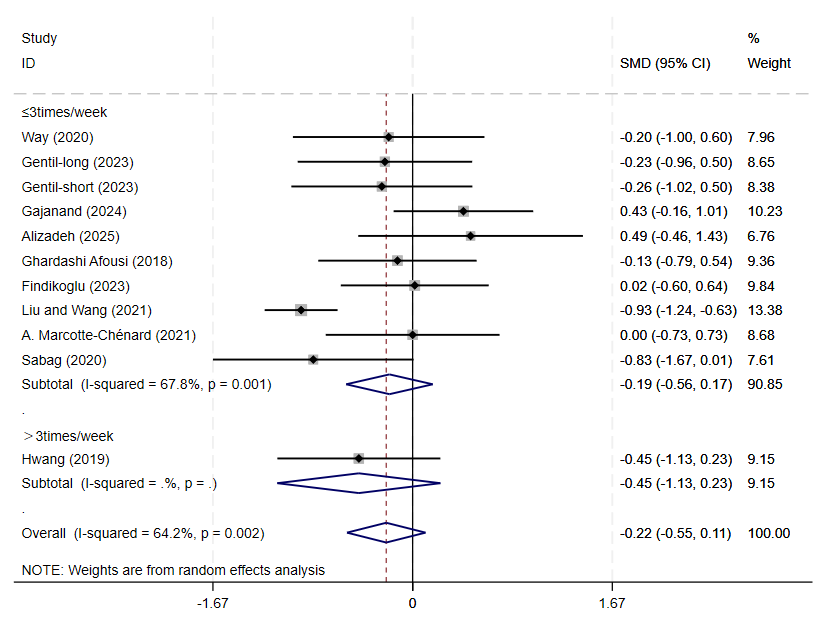


**Supplementary Figure 7.** Meta-analysis results on the effect of training frequency on TC in patients with type 2 diabetes


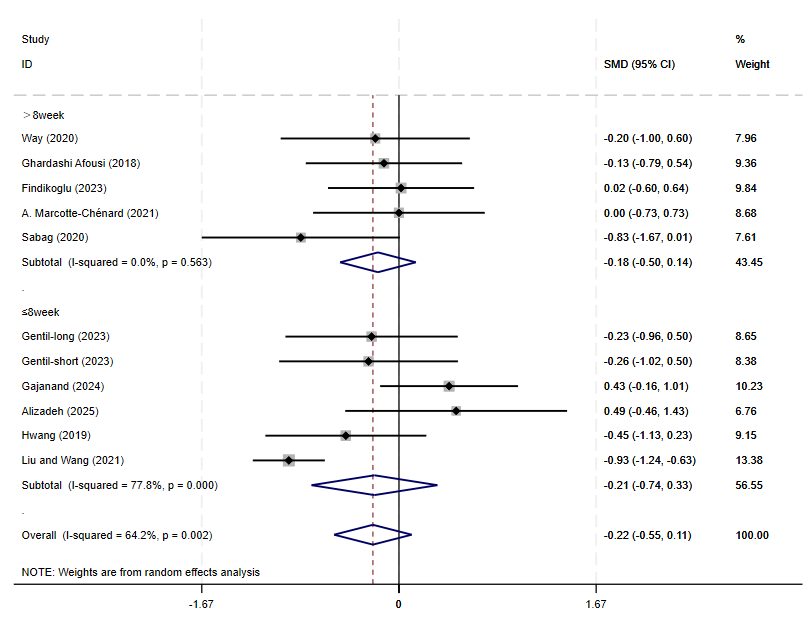


**Supplementary Figure 8.**Meta-analysis results on the effect of intervention duration on TC in patients with type 2 diabetes


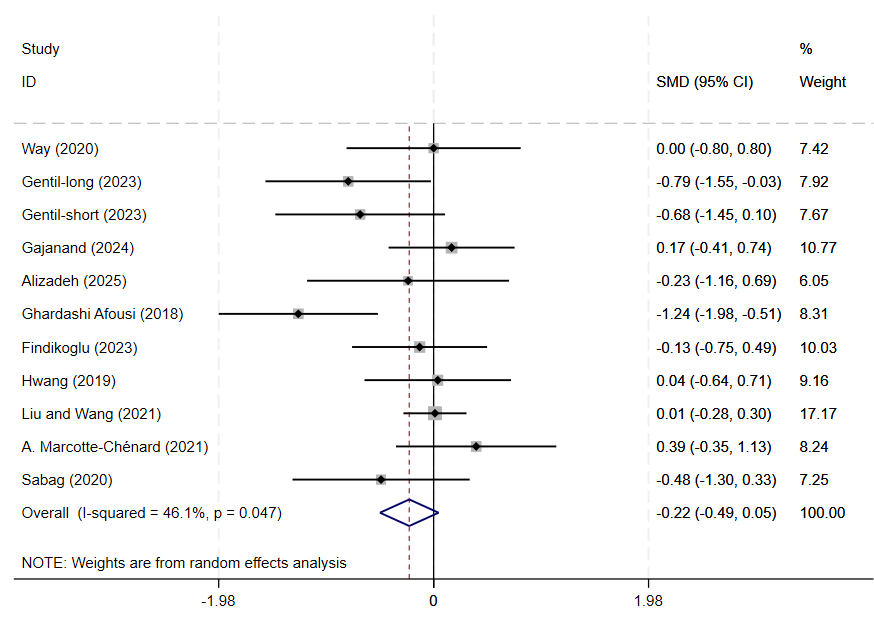
 **Supplementary Figure 9.** Meta-analysis of the effects of HIIT and MICT on TG in patients with type 2 diabetes.


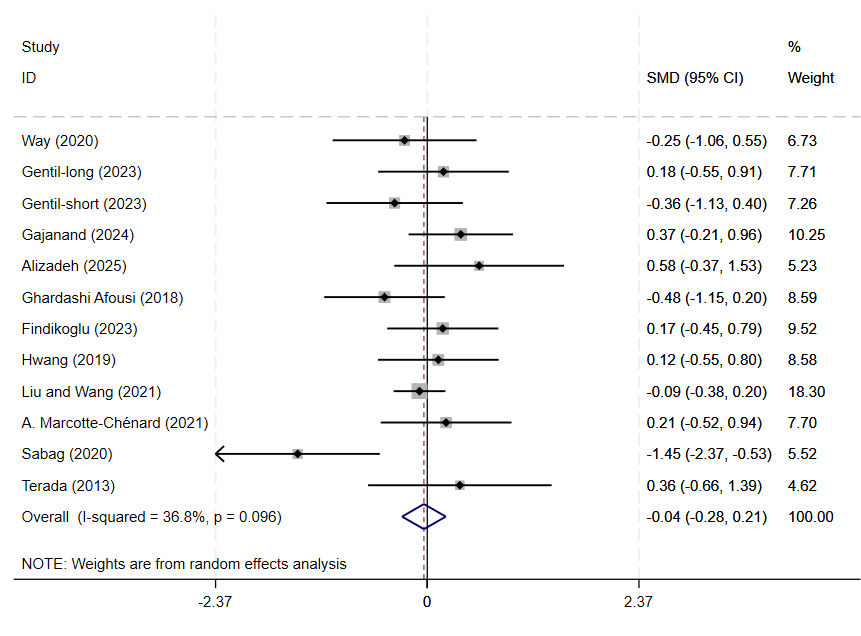
 **Supplementary Figure 10.** Meta-analysis of the effects of HIIT and MICT on LDL-C in patients with type 2 diabetes.


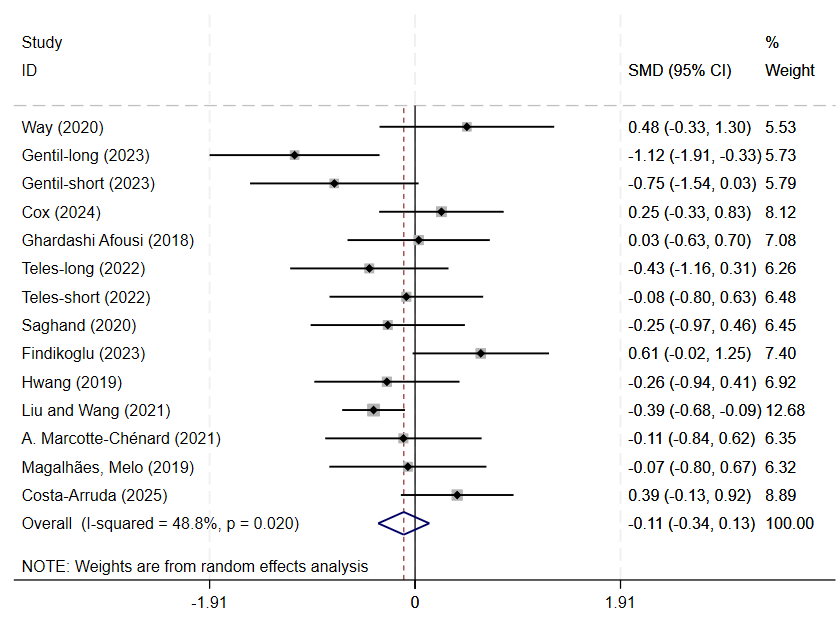
 **Supplementary Figure 11.** Meta-analysis of the effects of HIIT and MICT on SBP in patients with type 2 diabetes


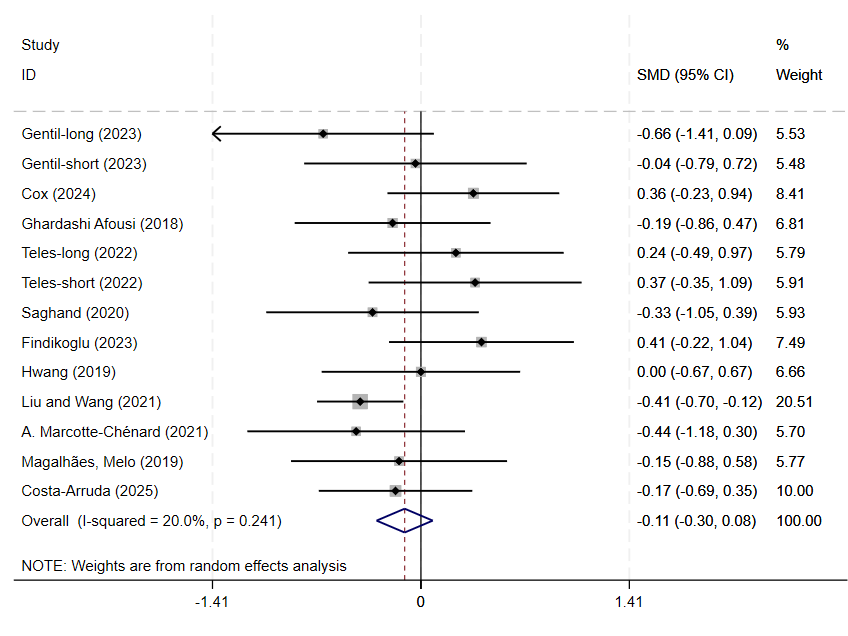
 **Supplementary Figure 12.** Meta-analysis of the effects of HIIT and MICT on DBP in patients with type 2 diabetes.


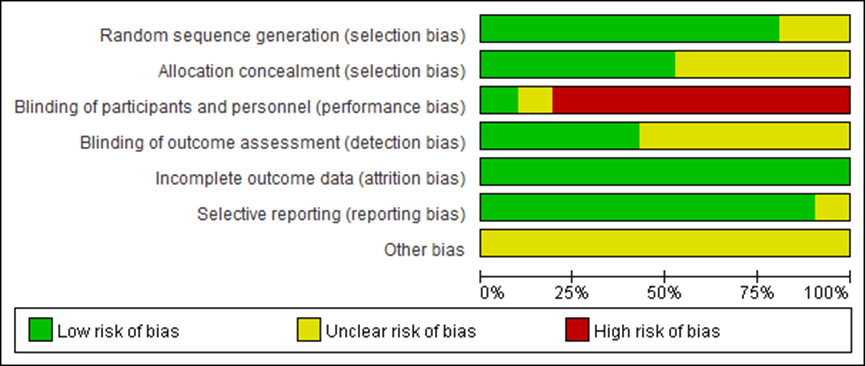


**Supplementary Figure 13.**Results of Cochrane risk of bias tool


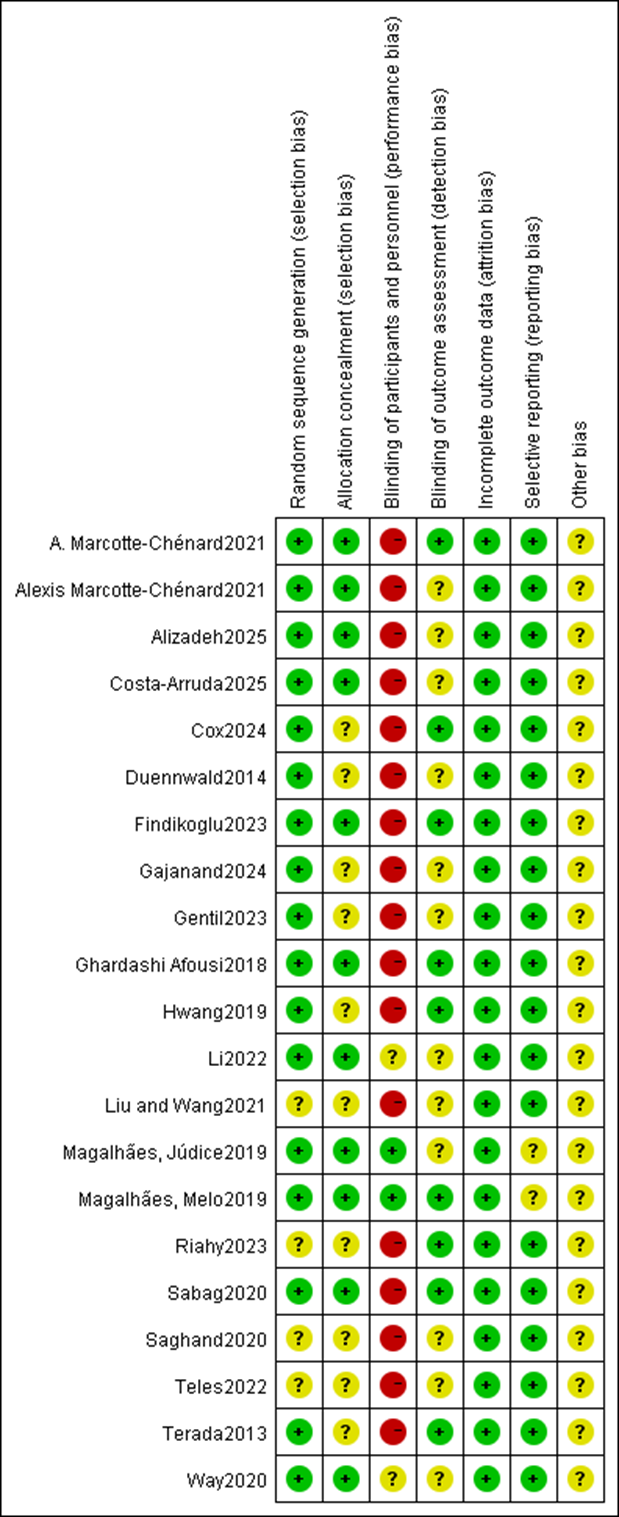


**Supplementary Figure 14.**Results of Cochrane risk of bias tool


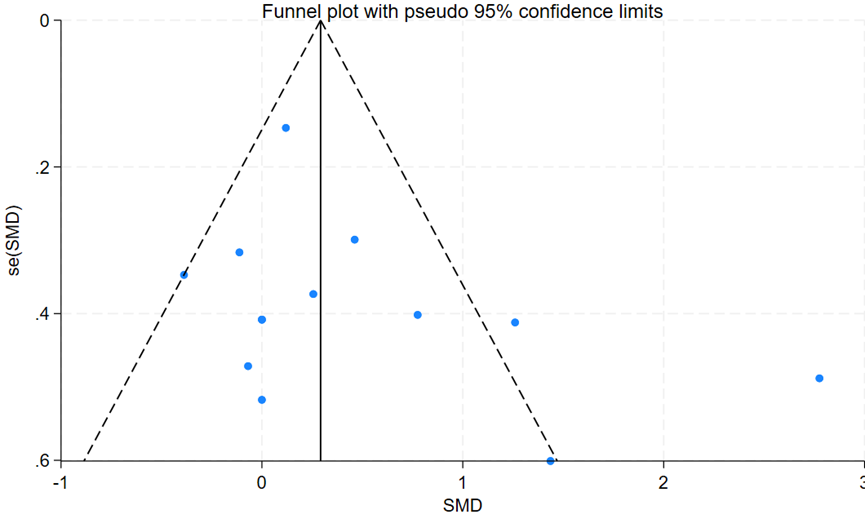


**Supplementary Figure 15.**Funnel plot（HDL)


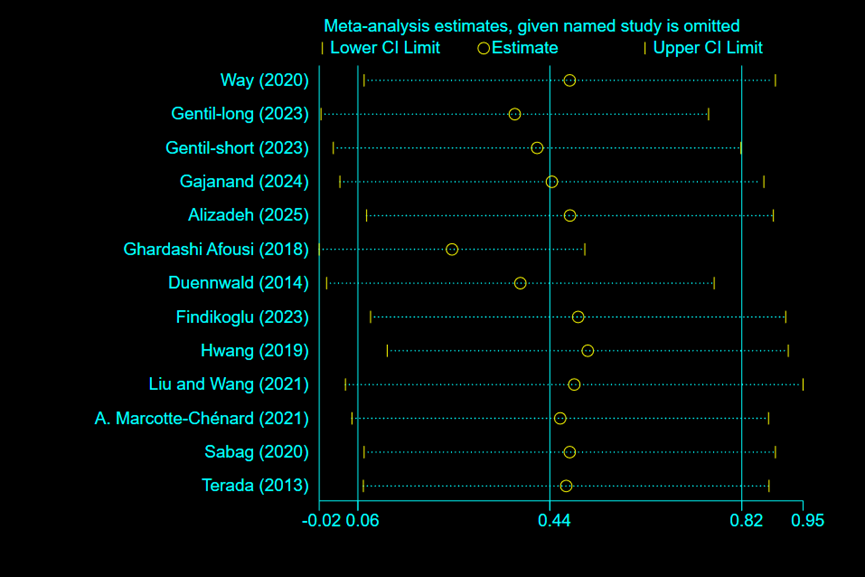


**Supplementary Figure 16.**Sensitivity analysis results (HDL)


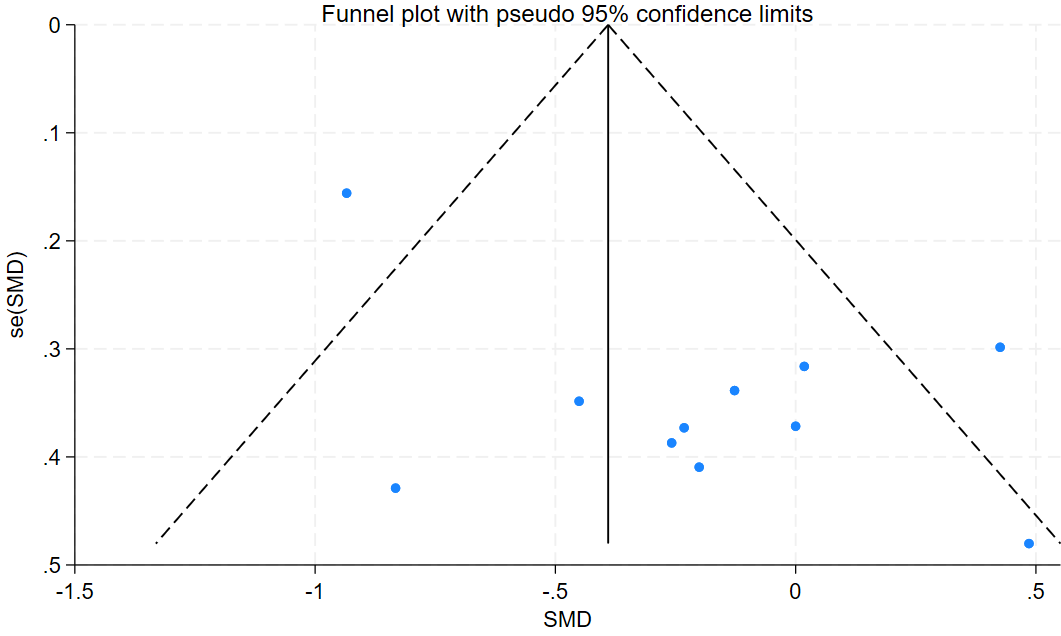


**Supplementary Figure 15.**Funnel plot(TC)


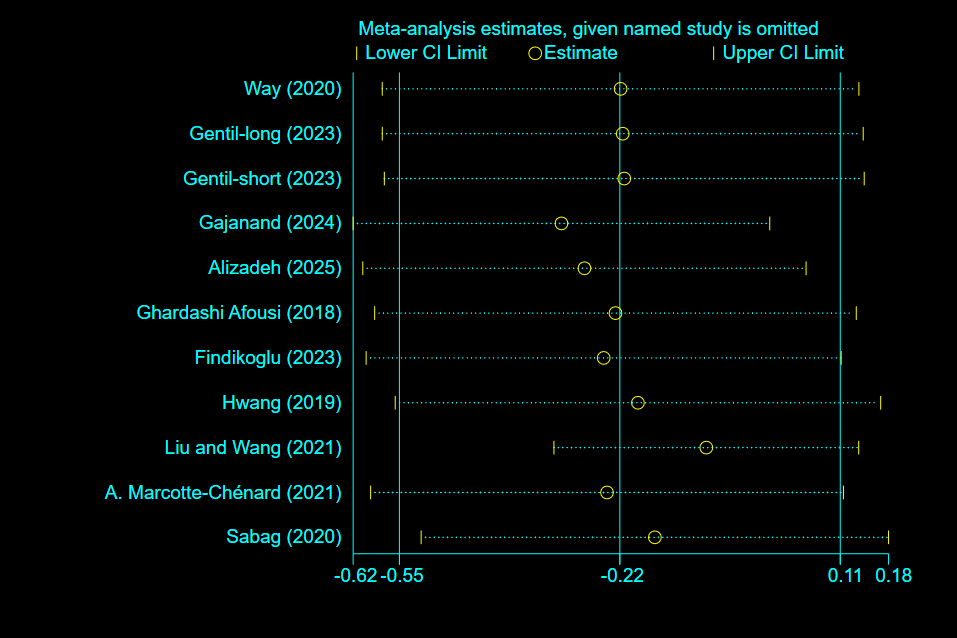


**Supplementary Figure 16.**Sensitivity analysis results (TC)

## Supplementary Tables

**Supplementary Table 1.**

| **Study** | **Year** | **Country** | **Sample size** | | **Gender**  **(M/F)** | **Mean age** | |
| --- | --- | --- | --- | --- | --- | --- | --- |
|  |  |  | **HIIT** | **MICT** |  | **HIIT** | **MICT** |
| Way | 2020 | Australia | 12 | 12 | 13/11 | 56.9(2.1) | 54.8(2.4) |
| Gentil | 2023 | Brazil | L-15  S-13 | 14 | 22/22 | L:57.3±8.9  S:55.7±7.4 | 54.6±8.9 |
| Gajanand | 2024 | Australia | 23 | 23 | 28/18 | 59.0±8.8 | 60.1±7.3 |
| Costa-Arruda | 2025 | Brazil | 30 | 27 | 18/39 | 55.3±12.58 | |
| **Alizadeh** | 2025 | Iran | 9 | 9 | 9/9 | 51.6±7.3 | 51.9±4.6 |
| **Cox** | 2024 | Australia | 23 | 23 | 28/18 | 59.0±8.8 | 60.1±7.3 |
| **Ghardashi Afousi** | 2018 | Iran | 18 | 17 | 16/19 | 54.78±6.19 | 53.12 ± 4.84 |
| **Alexis Marcotte-Chénard** | 2021 | Canada | 14 | 15 | 0/29 | 67.1(63.0–70.6) | 68.1(64.1-70.4) |
| **Teles** | 2022 | Brazil | L-14  S-15 | 15 | / | L:54.64±8.91  S:55.67±7.44 | 57.33±8.93 |
| **Li** | 2022 | China | 13 | 12 | 25/0 | 38±6 | 39±5 |
| **Saghand** | 2020 | Iran | 15 | 15 | 17/13 | 54.66±6.37 | 53.4±4.98 |
| **Duennwald** | 2014 | Australia | 8 | 7 | 11/4 | 59.6±2.0 | 59.6±2.3 |
| **Findikoglu** | 2023 | Turkey | 20 | 20 | 5/35 | 57.5±7.82 | 55.42±8.12 |
| **Hwang** | 2019 | America | 18 | 16 | 19/15 | 65±2 | 62±2 |
| **Liu and wang** | 2021 | China | 93 | 90 | 83/100 | 67.89±6.79 | 68.82±7.33 |
| **A.Marcotte-Chénard** | 2021 | Canada | 14 | 15 | 0/29 | 67.0±3.7 | 68.3±5.1 |
| **Riahy** | 2023 | Iran | 13 | 12 | 25/0 | 43.9±2.5 | 44.0±2.6 |
| **Sabag** | 2020 | Australia | 12 | 12 | 12/12 | 56.9(2.1) | 54.8(2.4) |
| **Terada** | 2013 | Canada | 8 | 7 | 8/7 | 62(3) | 63(5) |
| **Magalhães, Júdice** | 2019 | Portugal | 13 | 16 | 16/13 | 58.9±7.5 | 60.4±6.8 |
| **Magalhães, Melo** | 2019 | Portugal | 13 | 16 | 16/13 | 58.9±7.5 | 60.4±6.8 |

| **Study** | **session duration** | | **frequency** | | **Intervention cycle** | |
| --- | --- | --- | --- | --- | --- | --- |
|  | **HIIT** | **MICT** | **HIIT** | **MICT** | **HIIT** | **MICT** |
| **Way** | 19min | 55min | Three times a week | Three times a week | 12 weeks | 12 weeks |
| **Gentil** | L-24min S-24min | 18min | Two times a week | Two times a week | 8 weeks | 8 weeks |
| **Gajanand** | 26min | 52.5min | Three times a week | Three times a week | 8 weeks | 8 weeks |
| **Costa-Arruda** | 24min | 24min | / | / | / | / |
| **Alizadeh** | 36min | 45min | Three times a week | Three times a week | 8 weeks | 8 weeks |
| **Cox** | 26min | 52.5min | Three times a week | Four times a week | 8 weeks | 8 weeks |
| **Ghardashi Afousi** | 20min | 42min | Three times a week | Three times a week | 12 weeks | 12 weeks |
| **Alexis Marcotte-Chénard** | 25min | 50min | Three times a week | Three times a week | 12 weeks | 12 weeks |
| **Teles** | L-24min S-24min | 18min | Two times a week | Two times a week | 3 weeks | 3 weeks |
| **Li** | 15min | 30min | Five times a week | Five times a week | 12 weeks | 12 weeks |
| **Saghand** | 60min | 60min | / | / | 8 weeks | 8 weeks |
| **Duennwald** | 42min | 50.3min | Three times a week | Three times a week | 4 weeks | 4 weeks |
| **Findikoglu** | 34(1-4week)-  46(5-8week)-  58(9-12week) | 34(1-4week)-46(5-8week)-58(9-12week) | Three times a week | Three times a week | 12 weeks | 12 weeks |
| **Hwang** | 40min | 47min | Four times a week | Four times a week | 8 weeks | 8 weeks |
| **Liu and wang** | 20min | 50min | Three times a week | Three times a week | 8 weeks | 8 weeks |
| **A.Marcotte-Chénard** | 25min | 50min | Three times a week | Three times a week | 12 weeks | 12 weeks |
| **Riahy** | 40min | 62min | Three times a week | Three times a week | 12 weeks | 12 weeks |
| **Sabag** | 19min | 50-60min | Three times a week | Three times a week | 12 weeks | 12 weeks |
| **Terada** | 30(1-4week)-  45(5-8week)-  60(9-12week) | 30(1-4week)-  45(5-8week)-  60(9-12week) | Five times a week | Five times a week | 12 weeks | 12 weeks |
| **Magalhães, Júdice** | 50-55min | 60-65min | Three times a week | Three times a week | 12 months | 12 months |
| **Magalhães, Melo** | 50-55min | 60-65min | Three times a week | Three times a week | 12 months | 12 months |

| **Study** | **Outcome** | |
| --- | --- | --- |
|  | **HIIT** | **MICT** |
| **Way** | HbA1c(↓)、FBG(→)、BMI(→)、TC(→)、TG(→)、LDL(→)、HDL(→)、SBP(↓) | HbA1c(↓)、FBG(→)、BMI(→)、TC(→)、TG(→)、LDL(→)、HDL(→)、SBP(↓) |
| **Gentil** | Long:HbA1c(↓)、FBG(→)、TC(→)、TG(↓)、LDL(→)、HDL(→)、SBP(↓)、DBP(→)  Short:HbA1c(→)、FBG(→)、TC(→)、TG(→)、LDL(→)、HDL(→)、SBP(→)、DBP(→) | HbA1c(→)、FBG(→)、TC(→)、TG(→)、LDL(→)、HDL(→)、SBP(→)、DBP(→) |
| **Gajanand** | HbA1c(↓)、FBG(→)、HOMA-IR(→)、TC(→)、TG(→)、LDL(→)、HDL(↑)、 | HbA1c(↓)、FBG(→)、HOMA-IR(→)、TC(→)、TG(→)、LDL(→)、HDL(→)、 |
| **Costa-Arruda** | SBP(→)、DBP(→) | SBP(→)、DBP(→) |
| **Alizadeh** | HbA1c(→)、BMI(↓)、TC(→)、TG(→)、LDL(→)、HDL(→) | HbA1c(→)、BMI(↓)、TC(→)、TG(→)、LDL(→)、HDL(→) |
| **Cox** | HbA1c(↓)、FBG(→)、SBP(→)、DBP(→) | HbA1c(↓)、FBG(→)、SBP(→)、DBP(→) |
| **Ghardashi Afousi** | HOMA-IR(↓)、BMI(→)、TC(↓)、TG(↓)、LDL(↓)、HDL(↑)、SBP(↓)、DBP(↓) | HOMA-IR(↓)、BMI(→)、TC(↓)、TG(↓)、LDL(↓)、HDL(↑)、SBP(↓)、DBP(↓) |
| **Alexis Marcotte-Chénard** | HbA1c(→)、FBG(→)、HOMA2-IR(→)、BMI(→) | HbA1c(↓)、FBG(↓)、HOMA2-IR(→)、BMI(→) |
| **Teles** | Long:SBP(↓)、DBP(→)  Short:SBP(↓)、DBP(→) | SBP(→)、DBP(→) |
| **Li** | HbA1c(↓)、FBG(↓)、BMI(→) | HbA1c(↓)、FBG(↓)、BMI(↓) |
| **Saghand** | HbA1c(↓)、SBP(→)、DBP(↓) | HbA1c(↓)、SBP(→)、DBP(↓) |
| **Duennwald** | HbA1c(→)、FBG(→)、HOMA-IR(→)、BMI(→)、HDL(→) | HbA1c(→)、FBG(→)、HOMA-IR(→)、BMI(→)、HDL(→) |
| **Findikoglu** | HbA1c(↓)、HOMA-IR(→)、BMI(→)、TC(→)、TG(→)、LDL(→)、HDL(→)、SBP(→)、DBP(→) | HbA1c(↓)、HOMA-IR(→)、BMI(↓)、TC(→)、TG(→)、LDL(→)、HDL(→)、SBP(↓)、DBP(→) |
| **Hwang** | HbA1c(↓)、HOMA-IR(→)、BMI(→)、TC(→)、TG(→)、LDL(→)、HDL(→)、SBP(→)、DBP(→) | HbA1c(↓)、OMA-IR(→)、BMI(→)、TC(→)、TG(→)、LDL(→)、HDL(→)、SBP(→)、DBP(→) |
| **Liu and wang** | FBG(↓)、BMI(↓)、TC(↓)、TG(↓)、LDL(↓)、HDL(→)、SBP(↓)、DBP(↓) | FBG(↓)、BMI(→)、TC(↓)、TG(↓)、LDL(→)、HDL(→)、SBP(↓)、DBP(→) |
| **A.Marcotte-Chénard** | TC(→)、TG(→)、LDL(→)、HDL(→)、SBP(↓)、DBP(↓) | TC(→)、TG(→)、LDL(→)、HDL(→)、SBP(↓)、DBP(↓) |
| **Riahy** | HbA1c(↓)、HOMA-IR(↓)、BMI(↓) | HbA1c(↓)、HOMA-IR(↓)、BMI(↓) |
| **Sabag** | HbA1c(↓)、HOMA2-IR(→)、BMI(→)、TC(→)、TG(→)、LDL(→)、HDL(→) | HbA1c(↓)、HOMA2-IR(→)、BMI(→)、TC(→)、TG(→)、LDL(→)、HDL(→) |
| **Terada** | HbA1c(→)、FBG(→)、BMI(→)、LDL(→)、HDL(→) | HbA1c(→)、FBG(→)、BMI(→)、LDL(→)、HDL(→) |
| **Magalhães, Júdice** | HOMA2-IR(→)、BMI(→)、 | HOMA2-IR(→)、BMI(↓)、 |
| **Magalhães, Melo** | SBP(→)、DBP(→) | SBP(→)、DBP(→) |

Note：→ no significance; ↑ increased significance; ↓ decreased significance

| **Study** | **Exercise Mode** | **Intensity Parameters** | |
| --- | --- | --- | --- |
|  |  | **HIIT** | **MICT** |
| **Way** | Upright cycle ergometer | 1 × 4 min high-intensity bout at a workload designed to elicit 90% VO2peak, three days a week. Each session included a ten-minute warm-up at 50%VO2peak and a five- minute cool-down at 50% VO2peak | 45 min of continuous cycling on the ergometer at an intensity of 60% VO2peak, Each session included a ten-minute warm-up at 50%VO2peak and a five- minute cool-down at 50% VO2peak |
| **Gentil** | motorized treadmill | L-HIIT:5 bouts of 2 minutes walking/running at 100% of v _VO2max  S-HIIT: 20 bouts of 30 seconds walking/running at 100% of v_VO2max | 70% of v _VO2max |
| **Gajanand** | Treadmill/cycle + resistance training | Each session consisted of  a 3-min aerobic warm-up before 1×4min of high-intensity aerobic exercise (85%–95% of Hrpeak)  RPE≥17 | participants completed 22.5min of aerobic exercise (55%–69%  of HRpeak) followed by 30min of resistance-type exercises (RPE of 11–13 (fairly light to somewhat hard)) |
| **Costa-Arruda** | Motorized treadmill | 85–100% HRmax； active recovery 60s | 60–70% HRmax |
| **Alizadeh** | Treadmill | Warm-up 60–70% HRR; Main 90–95% HRR;  recovery 60–70% HRR | Warm-up 40–50% HRR; main 60–70% HRR |
| **Cox** | Cycle ergometer | Warm-up 50–60% of HR peak;  Main 85–95% HR peak; recovery 50–60% of HR peak  RPE ≥ 17 | 55–69% of HRpeak  RPE 11–13 |
| **Ghardashi Afousi** | Motorized treadmill | 12 intervals of 1.5 min at 85%-90% HRmax and 2 min at 55%-60%  HRmax | 70% HRmax |
| **Alexis Marcotte-Chénard** | treadmill | warm-up：2 min at 55% HRR and 1 min at 75% HRR  Main：90% HRR  Recovery：45% HRR | warm-up：45% HRR  main：60% HRR  Recovery：40% HRR |
| **Teles** | treadmill | long HIIE：2 min at 100% v ˙Vo2peak + 2 min of  passive rest  short HIIE：30 s at 100% v ˙Vo2peak + 30 s of passive rest | 70%v ˙Vo2peak |
| **Li** | Power cycling | HIIT：80%–95%VO2max  passive or active rest (25%–30% VO2max) | 50%–70% VO2max |
| **Saghand** | cycle ergometer | Warm-up 40% HRmax;  Main training 80–90% HRmax;  Interval：50-60%Hrmax  recovery 40% HRmax | Warm-up 40% HRmax; Main 60–70% HRmax; recovery 40% HRmax; |
| **Duennwald** | bicycle ergometer | Warm-up 70% HRmax;  Main training 90–95% HRmax;  recovery 70% HRmax | 70% HRmax |
| **Findikoglu** | Ergoline | trained with HIIT at 90 and 30% of their VO2peak (1:2 minratio) | 50% of VO2peak |
| **Hwang** | Airdyne AD4 | Warm-up 70% HRpeak ;  Main 90% HRpeak;  recovery 70% HRpeak | 70% HRpeak |
| **Liu and wang** | cycle ergometer | participants performed  20 repetitions of high-intensity interval exercise(50 s cycling at  90% of HRmax and 10 s passive recovery at 20% of HRmax) on a cycle  ergometer for 20 min | participants performed 50-min of continuous exercise at an intensity of ~65% HRmax |
| **A.Marcotte-Chénard** | treadmill | warm-up：2 min at 55% HRR and 1 min at 75% HRR  Main：90% HRR  Recovery：45% HRR | warm-up：45% HRR  main：60% HRR  Recovery：40% HRR |
| **Riahy** | walking/running | involved 4×4minute walked or ran at  85-95% maximum heart rate (HRmax), with three  minutes of active rest at 50–60% HRmax in between. | walking/running continuously for  47min at 60–70% HRmax |
| **Sabag** | cycling | Each exercise session included 4 min of cycling at a work rate equivalent  to 90% VO2peak and a 10-min warm-up and 5-min cool-down at a work rate  equivalent to 50% VO2peak | The exercise duration was progressed from  30 min in the 1st week to 45 min by the 4th week and involved continuous cycling at a work rate equivalent to 60%  VO2peak. |
| **Terada** | Stationary cycling and treadmill walking | 100% VO₂R;recovery intervals at 20% VO2R | 40% VO2R |
| **Magalhães, Júdice** | cycling | Phase 1（1–4week）：40–60% HRR  Phase 2（5–8week）：2min 70–80% HRR + 1min 40–60% HRR  Phase 3（9–52week）：1min 90% HRR + 1min40–60% HRR | 40–60% HRR |
| **Magalhães, Melo** | cycling | Phase 1（1–4week）：40–60% HRR  Phase 2（5–8week）：2min 70–80% HRR + 1min 40–60% HRR  Phase 3（9–52week）：1min 90% HRR + 1min40–60% HRR | 40–60% HRR |

| Study | **Cardiorespiratory Fitness** | | **Diabetes duration (y)** | | **Smoking Status** | |
| --- | --- | --- | --- | --- | --- | --- |
|  | **HIIT** | **MICT** | **HIIT** | **MICT** | **HIIT** | **MICT** |
| Way | 20.9(2.6)  V̇O₂peak | 21.6 (6.0)  V̇O₂peak | 9.3 (7.3) | 8.2 (5.8) | NR | NR |
| Gentil | 22.6±8.9  V̇O₂max (ml/kg/min) | 22.4±5.6  V̇O₂max (ml/kg/min) | NR | NR | 0(0％) | |
| Gajanand | NR | NR | 9.2±7.4 | 10.6±8.4 | Ex-smoker  5 (21.7％)  Never  18 (78.3％) | Ex-smoker  5 (21.7％)  Never  18 (78.3％) |
| Costa-Arruda | NR | NR | 10.16 ± 8.83 | | Ex-smoking 17 (30％) | |
| Alizadeh | NR | NR | 6.7±5.1 | 6.7±5.1 | NR | NR |
| Cox | 24.3±5.1  V̇O₂peak | 24.7±7.0  V̇O₂peak | 9.2±7.4 | 10.6±8.4 | NR | NR |
| Ghardashi Afousi | NR | NR | 8.67±2.40 | 9 ± 2.39 | 0(0.0％) | 0（0.0％） |
| Alexis Marcotte-Chénard | 1.4 ± 0.2  VO₂peak (mL/min) | 1.4 ± 0.3  VO₂peak (mL/min) | NR | NR | 0(0.0％) | 0（0.0％） |
| Teles | NR | NR | NR | NR | NR | NR |
| Li | 3.39±0.44  VO₂max (L/min) | 3.46±0.38  VO₂max (L/min) | 1.95±0.55 | 1.79±0.52 | 5(38.5％) | 5(41.7％) |
| Saghand | 22.73 ± 4.21  VO₂max (mL/kg/min) | 22.80 ± 3.86  VO₂max (mL/kg/min) | 3.93±1.38 | 3.86±1.35 | 0(0.0％) | 0（0.0％） |
| Duennwald | 28.3 ± 1.9  VO₂peak (mL/kg/min) | 26.6 ± 2.0  VO₂peak (mL/kg/min) | 11 ± 2 | 7 ± 2 | 0(0.0％) | 0（0.0％） |
| Findikoglu | 16.09 ± 2.79  VO₂peak (mL/kg/min) | 15.25 ± 2.15  VO₂peak (mL/kg/min) | NR | NR | 0(0.0％) | 0（0.0％） |
| Hwang | 2.06 ± 0.15  VO₂peak (L/min) | 1.96 ± 0.12  VO₂peak (L/min) | 7.8 ± 1.3 | 8.3 ± 1.5 | 0(0.0％) | 0（0.0％） |
| Liu and wang | 31.89±3.03 VO₂max (mL/min/kg) | 32.11±3.27 VO₂max (mL/min/kg) | 9.79 ± 8.38 | 10.38 ± 7.93 | 28.1% | 28.3% |
| A.Marcotte-Chénard | 1.4 ± 0.2  VO₂peak (mL/min) | 1.4 ± 0.3  VO₂peak (mL/min) | NR | NR | 0(0.0％) | 0（0.0％） |
| Riahy | 28.6 ± 2.9  VO₂peak (mL·kg⁻¹·min⁻¹) | 30.1 ± 2.2  VO₂peak (mL·kg⁻¹·min⁻¹) | NR | NR | NR | NR |
| Sabag | 20.9 ± 0.7  VO₂peak (mL/kg/min) | 21.6 ± 1.7  VO₂peak (mL/kg/min) | 9.3 ± 2.1 | 8.2 ± 1.7 | NR | NR |
| Terada | 22.8 (5.4)  VO2peak (ml/kg/min) | 18.1 (2.7)  VO2peak (ml/kg/min) | 6 (4) | 8 (4) | 0(0.0％) | 0（0.0％） |
| Magalhães, Júdice | 27.1±6.3  VO2max (ml/kg/min) | 24.1±3.2  VO2max (ml/kg/min) | 5.7±3.7 | 8.4±5.1 | NR | NR |
| Magalhães, Melo | 27.1±6.3  VO2max (ml/kg/min) | 24.1±3.2  VO2max (ml/kg/min) | 5.7±3.7 | 8.4±5.1 | NR | NR |

| Study | **Comorbidities** | | **Medications** | |
| --- | --- | --- | --- | --- |
|  | **HIIT** | **MICT** | **HIIT** | **MICT** |
| Way | NR | NR | Anti-Hyperglycemic:12(100％)  Anti-Hypertensive:8(66.7％)  Lipid Lowering:5(41.7％) | Anti-  Hyperglycemic:12(100％)  Anti-Hypertensive:8(66.7％)  Lipid Lowering:5(41.7％) |
| Gentil | NR | NR | Biguanides 11(73.3％)  Sulfonylureas 1(6.7％)  SGLT2 inhibitors 2(13.3％)  DPP-4 inhibitors 1(6.7％)  GLP-1 analogue 0  Pioglitazones 0  Insulin 8(53.3％)  Anti-Hypertensive 15(100％)  Anticholesterolemic 13(86.7％) | Biguanides 10(66.7％)  Sulfonylureas 3(20％)  SGLT2 inhibitors 1(6.7％)  DPP-4 inhibitors 1(6.7％)  GLP-1 analogue 2(13.3％)  Pioglitazones 1(6.7％)  Insulin 5(33.3％)  Anti-Hypertensive 14(93.3％)  Anticholesterolemic 11(73.3％) |
| Gajanand | NR | NR | Oral antihyperglycaemics:  20 (87%);  Insulin: 4 (17.4%) | Oral antihyperglycaemics:  20 (87%);  Insulin: 3 (13.0%) |
| Costa-Arruda | Arterial hypertension  53 (93％)  Dyslipidemia  34 (60％)  Ex-smoking  17 (30％) | | oral antidiabetic:57 (100)  Insulin:25 (44)  Antihypertensive:53 (93) | |
| Alizadeh | NR | NR | Metformin:8 (88.9%)  Sulfonylurea:5 (55.6%)  DPP-4 Inhibitors  3 (33.3%)  Anti-Diabetes:8 (88.9%)  Statins:4 (44.4%)  Fenofibrate:1 (11.1%)  Anti-Lipids:5 (55.6%) | Metformin :9 (100%)  Sulfonylurea:3 (33.3%)  DPP-4 Inhibitors  2 (22.2%)  Anti-Diabetes:9 (100.0%)  Statins :7 (77.8%)  Fenofibrate:0 (0.0%)  Anti-Lipids:7 (77.8%) |
| Cox | NR | NR | Oral Antihyperglycaemics 20 (87.0％)  Insulin4 (17.4％)  Anti-hypertensives  17 (73.9％)  Statins, 13 (56.5％) | Oral Antihyperglycaemics 20 (87.0％)  Insulin3 (13.0％)  Anti-hypertensives  20 (87.0％)  Statins15 (65.2％) |
| Ghardashi Afousi | NR | NR | Diuretic 9 (50%)  ACE inhibitors 4 (22%)  Angiotensin blockers  3 (17%)  Metformin 9 (50%)  Sulfonylureas 8 (44%)  DPP-4 inhibitors  6 (33%)  Statins 8 (44%) | Diuretic 8 (47%)  ACE inhibitors 5 (29%)  Angiotensin blockers  2 (12%)  Metformin 11 (65%)  Sulfonylureas 5 (29%)  DPP-4 inhibitors  6 (35%)  Statins 5 (29%) |
| Alexis Marcotte-Chénard | NR | NR | Metformin 10 (71％)  Sulfonylurea 2 (14％)  DPP-4 inhibitor 6 (43％)  SGLT2 inhibitor 2 (14％) Thiazolidinediones  1 (7％)  Calcium channel blocker 2 (14％)  ACE inhibitor 11 (79％) Diuretic 7 (47％)  Beta blocker 4 (29％)  Statin 12 (86％) | Metformin 10 (67％)  Sulfonylurea 4 (27％)  DPP-4 inhibitor 6 (40％)  SGLT2 inhibitor 1 (7％) Thiazolidinediones  0 (0％)  Calcium channel blocker 4 (27％)  ACE inhibitor 13 (87％) Diuretic 6 (43％)  Beta blocker 2 (13％)  Statin 10 (67％) |
| Teles | NR | NR | NR | NR |
| Li | 3  (23.1) | 4  (33.3％) | Metformin 6(46.1％)  Sulfonylureas 3(23.1％)  DPP-4 inhibitors 3(23.1％)  Alpha-glucosidase inhibitor 1(7.7％) | Metformin 6(50％)  Sulfonylureas 2(16.7％)  DPP-4 inhibitors 2(16.7％)  Alpha-glucosidase inhibitor 0(0.0％) |
| Saghand | NR | NR | Metformin consumption  9 (60%)  Sulfonylureaconsumption  6 (40%)  Captopril consumption  4 (26%)  Atorvastatinconsumption  8 (53%) | Metformin consumption  8 (53%)  Sulfonylureaconsumption  5 (33%)  Captopril consumption  3 (20%)  Atorvastatinconsumption  8 (53%) |
| Duennwald | NR | NR | NR | NR |
| Findikoglu | NR | NR | NR | NR |
| Hwang | NR | NR | Metformin 10 (56％)  SGLT2 inhibitors 2 (11％)  Sulfonylureas 4 (22％)  DPP-4 inhibitors 2 (11％)  GLP-1 agonists 1 (6％)  Thiazolidinediones 3 (17％)  Insulin 4 (22％)  Statins 13 (72％)  Anti-hypertensives 12 (67％)  Aspirin 8 (44％) | Metformin 10 (63％)  SGLT2 inhibitors 2 (13％)  Sulfonylureas 2 (13％)  DPP-4 inhibitors 5 (31％)  GLP-1 agonists 0 (0％)  Thiazolidinediones 0 (0％)  Insulin 4 (25％)  Statins 13 (81％)  Anti-hypertensives 12 (75％)  Aspirin 6 (38％) |
| Liu and wang | NR | NR | NR | NR |
| A.Marcotte-Chénard | NR | NR | Metformin 10 (71％)  Sulfonylurea 2 (14％)  DPP-4 inhibitor 6 (43％)  SGLT2 inhibitor 2 (14％) Thiazolidinediones  1 (7％)  Calcium channel blocker 2 (14％)  ACE inhibitor 11 (79％) Diuretic 7 (47％)  Beta blocker 4 (29％)  Statin 12 (86％) | Metformin 10 (67％)  Sulfonylurea 4 (27％)  DPP-4 inhibitor 6 (40％)  SGLT2 inhibitor 1 (7％) Thiazolidinediones  0 (0％)  Calcium channel blocker 4 (27％)  ACE inhibitor 13 (87％) Diuretic 6 (43％)  Beta blocker 2 (13％)  Statin 10 (67％) |
| Riahy | NR | NR | NR | NR |
| Sabag | NR | NR | NR | NR |
| Terada | NR | NR | NR | NR |
| Magalhães, Júdice | NR | NR | NR | NR |
| Magalhães, Melo | NR | NR | NR | NR |

Note：NR , No reports

**Supplementary Table 2.**

| Results | Search strategy | Database name/ Date of search |
| --- | --- | --- |
| 526 | TS=(Diabetes Mellitus, Type 2) OR TS=(Diabetes Mellitus, Stable) OR TS=(Stable Diabetes Mellitus) OR TS=(Diabetes Mellitus, Noninsulin Dependent) OR TS=(Diabetes Mellitus, Adult-Onset) OR TS=(Adult-Onset Diabetes Mellitus) OR TS=(Diabetes Mellitus, Adult Onset) OR TS=(Diabetes Mellitus, Ketosis-Resistant) OR TS=(Diabetes Mellitus, Ketosis Resistant) OR TS=(Ketosis-Resistant Diabetes Mellitus) OR TS=(Diabetes Mellitus, Non Insulin Dependent) OR TS=(Diabetes Mellitus, Non-Insulin-Dependent) OR TS=(Non-Insulin-Dependent Diabetes Mellitus) OR TS=(Diabetes Mellitus, Type II) OR TS=(NIDDM) OR TS=(Diabetes Mellitus, Maturity-Onset) OR TS=(Diabetes Mellitus, Maturity Onset) OR TS=(Maturity-Onset Diabetes Mellitus) OR TS=(Maturity Onset Diabetes Mellitus) OR TS=(MODY) OR TS=(Diabetes Mellitus, Slow-Onset) OR TS=(Diabetes Mellitus, Slow Onset) OR TS=(Slow-Onset Diabetes Mellitus) OR TS=(Type 2 Diabetes Mellitus) OR TS=(Noninsulin-Dependent Diabetes Mellitus) OR TS=(Noninsulin Dependent Diabetes Mellitus) OR TS=(Maturity-Onset Diabetes) OR TS=(Diabetes, Maturity-Onset) OR TS=(Maturity Onset Diabetes) OR TS=(Type 2 Diabetes) OR TS=(Diabetes, Type 2) OR TS=(Diabetes Mellitus, Noninsulin-Dependent) AND TS=(High-Intensity Interval Training) OR TS=(High Intensity Interval Training) OR TS=(High-Intensity Interval Trainings) OR TS=(Interval Training, High-Intensity) OR TS=(Interval Trainings, High-Intensity) OR TS=(Training, High-Intensity Interval) OR TS=(Trainings, High-Intensity Interval) OR TS=(High-Intensity Intermittent Exercise) OR TS=(Exercise, High-Intensity Intermittent) OR TS=(Exercises, High-Intensity Intermittent) OR TS=(High-Intensity Intermittent Exercises) OR TS=(Sprint Interval Training) OR TS=(Sprint Interval Trainings) | Web of Science/ 14 November 2025 |
| 406 | MeSH descriptor: [Diabetes Mellitus, Type 2] explode all trees OR (Diabetes Mellitus, Type 2):ti,ab,kw OR (Diabetes Mellitus, Stable):ti,ab,kw OR (Stable Diabetes Mellitus):ti,ab,kw OR (Diabetes Mellitus, Noninsulin Dependent):ti,ab,kw OR (Diabetes Mellitus, Adult-Onset):ti,ab,kw OR (Adult-Onset Diabetes Mellitus):ti,ab,kw OR (Diabetes Mellitus, Adult Onset):ti,ab,kw OR (Diabetes Mellitus, Ketosis-Resistant):ti,ab,kw OR (Diabetes Mellitus, Ketosis Resistant):ti,ab,kw OR (Ketosis-Resistant Diabetes Mellitus):ti,ab,kw OR (Diabetes Mellitus, Non Insulin Dependent):ti,ab,kw OR (Diabetes Mellitus, Non-Insulin-Dependent):ti,ab,kw OR (Non-Insulin-Dependent Diabetes Mellitus):ti,ab,kw OR (Diabetes Mellitus, Type II):ti,ab,kw OR (NIDDM):ti,ab,kw OR (Diabetes Mellitus, Maturity-Onset):ti,ab,kw OR (Diabetes Mellitus, Maturity Onset):ti,ab,kw OR (Maturity-Onset Diabetes Mellitus):ti,ab,kw OR (Maturity Onset Diabetes Mellitus):ti,ab,kw OR (MODY):ti,ab,kw OR (Diabetes Mellitus, Slow-Onset):ti,ab,kw OR (Diabetes Mellitus, Slow Onset):ti,ab,kw OR (Slow-Onset Diabetes Mellitus):ti,ab,kw OR (Type 2 Diabetes Mellitus):ti,ab,kw OR (Noninsulin-Dependent Diabetes Mellitus):ti,ab,kw OR (Noninsulin Dependent Diabetes Mellitus):ti,ab,kw OR (Maturity-Onset Diabetes):ti,ab,kw OR (Diabetes, Maturity-Onset):ti,ab,kw OR (Maturity Onset Diabetes):ti,ab,kw OR (Type 2 Diabetes):ti,ab,kw OR (Diabetes, Type 2):ti,ab,kw OR (Diabetes Mellitus, Noninsulin-Dependent):ti,ab,kw AND MeSH descriptor: [High-Intensity Interval Training] explode all trees OR (High-Intensity Interval Training):ti,ab,kw OR (High Intensity Interval Training):ti,ab,kw OR (High-Intensity Interval Trainings):ti,ab,kw OR (Interval Training, High-Intensity):ti,ab,kw OR (Interval Trainings, High-Intensity):ti,ab,kw OR (Training, High-Intensity Interval):ti,ab,kw OR (Trainings, High-Intensity Interval):ti,ab,kw OR (High-Intensity Intermittent Exercise):ti,ab,kw OR (Exercise, High-Intensity Intermittent):ti,ab,kw OR (Exercises, High-Intensity Intermittent):ti,ab,kw OR (High-Intensity Intermittent Exercises):ti,ab,kw OR (Sprint Interval Training):ti,ab,kw OR (Sprint Interval Trainings):ti,ab,kw | Cochrane/ 14 November 2025 |
| 357 | Search:((Diabetes Mellitus, Type 2[MeSH Terms]) OR ((((((((((((((((((((((((((((((((Diabetes Mellitus, Type 2[Title/Abstract]) OR (Diabetes Mellitus, Stable[Title/Abstract])) OR (Stable Diabetes Mellitus[Title/Abstract])) OR (Diabetes Mellitus, Noninsulin Dependent[Title/Abstract])) OR (Diabetes Mellitus, Adult-Onset[Title/Abstract])) OR (Adult-Onset Diabetes Mellitus[Title/Abstract])) OR (Diabetes Mellitus, Adult Onset[Title/Abstract])) OR (Diabetes Mellitus, Ketosis-Resistant[Title/Abstract])) OR (Diabetes Mellitus, Ketosis Resistant[Title/Abstract])) OR (Ketosis-Resistant Diabetes Mellitus[Title/Abstract])) OR (Diabetes Mellitus, Non Insulin Dependent[Title/Abstract])) OR (Diabetes Mellitus, Non-Insulin-Dependent[Title/Abstract])) OR (Non-Insulin-Dependent Diabetes Mellitus[Title/Abstract])) OR (Diabetes Mellitus, Type II[Title/Abstract])) OR (NIDDM[Title/Abstract])) OR (Diabetes Mellitus, Maturity-Onset[Title/Abstract])) OR (Diabetes Mellitus, Maturity Onset[Title/Abstract])) OR (Maturity-Onset Diabetes Mellitus[Title/Abstract])) OR (Maturity Onset Diabetes Mellitus[Title/Abstract])) OR (MODY[Title/Abstract])) OR (Diabetes Mellitus, Slow-Onset[Title/Abstract])) OR (Diabetes Mellitus, Slow Onset[Title/Abstract])) OR (Slow-Onset Diabetes Mellitus[Title/Abstract])) OR (Type 2 Diabetes Mellitus[Title/Abstract])) OR (Noninsulin-Dependent Diabetes Mellitus[Title/Abstract])) OR (Noninsulin Dependent Diabetes Mellitus[Title/Abstract])) OR (Maturity-Onset Diabetes[Title/Abstract])) OR (Diabetes, Maturity-Onset[Title/Abstract])) OR (Maturity Onset Diabetes[Title/Abstract])) OR (Type 2 Diabetes[Title/Abstract])) OR (Diabetes, Type 2[Title/Abstract])) OR (Diabetes Mellitus, Noninsulin-Dependent[Title/Abstract]))) AND ((High-Intensity Interval Training[MeSH Terms]) OR (((((((((((((High-Intensity Interval Training[Title/Abstract]) OR (High Intensity Interval Training[Title/Abstract])) OR (High-Intensity Interval Trainings[Title/Abstract])) OR (Interval Training, High-Intensity[Title/Abstract])) OR (Interval Trainings, High-Intensity[Title/Abstract])) OR (Training, High-Intensity Interval[Title/Abstract])) OR (Trainings, High-Intensity Interval[Title/Abstract])) OR (High-Intensity Intermittent Exercise[Title/Abstract])) OR (Exercise, High-Intensity Intermittent[Title/Abstract])) OR (Exercises, High-Intensity Intermittent[Title/Abstract])) OR (High-Intensity Intermittent Exercises[Title/Abstract])) OR (Sprint Interval Training[Title/Abstract])) OR (Sprint Interval Trainings[Title/Abstract]))) | Pubmed/ 16 November 2025 |
| 683 | ('non insulin dependent diabetes mellitus'/exp OR 'diabetes mellitus, type 2':ab,ti OR 'diabetes mellitus, stable':ab,ti OR 'stable diabetes mellitus':ab,ti OR 'diabetes mellitus, noninsulin dependent':ab,ti OR 'diabetes mellitus, adult-onset':ab,ti OR 'adult-onset diabetes mellitus':ab,ti OR 'diabetes mellitus, adult onset':ab,ti OR 'diabetes mellitus, ketosis-resistant':ab,ti OR 'diabetes mellitus, ketosis resistant':ab,ti OR 'ketosis-resistant diabetes mellitus':ab,ti OR 'diabetes mellitus, non insulin dependent':ab,ti OR 'diabetes mellitus, non-insulin-dependent':ab,ti OR 'non-insulin-dependent diabetes mellitus':ab,ti OR 'diabetes mellitus, type ii':ab,ti OR 'niddm':ab,ti OR 'diabetes mellitus, maturity-onset':ab,ti OR 'diabetes mellitus, maturity onset':ab,ti OR 'maturity-onset diabetes mellitus':ab,ti OR 'maturity onset diabetes mellitus':ab,ti OR 'mody':ab,ti OR 'diabetes mellitus, slow-onset':ab,ti OR 'diabetes mellitus, slow onset':ab,ti OR 'slow-onset diabetes mellitus':ab,ti OR 'type 2 diabetes mellitus':ab,ti OR 'noninsulin-dependent diabetes mellitus':ab,ti OR 'noninsulin dependent diabetes mellitus':ab,ti OR 'maturity-onset diabetes':ab,ti OR 'diabetes, maturity-onset':ab,ti OR 'maturity onset diabetes':ab,ti OR 'type 2 diabetes':ab,ti OR 'diabetes, type 2':ab,ti OR 'diabetes mellitus, noninsulin-dependent':ab,ti) AND ('high intensity interval training'/exp OR 'high-intensity interval training':ab,ti OR 'high intensity interval training':ab,ti OR 'high-intensity interval trainings':ab,ti OR 'interval training, high-intensity':ab,ti OR 'interval trainings, high-intensity':ab,ti OR 'training, high-intensity interval':ab,ti OR 'trainings, high-intensity interval':ab,ti OR 'high-intensity intermittent exercise':ab,ti OR 'exercise, high-intensity intermittent':ab,ti OR 'exercises, high-intensity intermittent':ab,ti OR 'high-intensity intermittent exercises':ab,ti OR 'sprint interval training':ab,ti OR 'sprint interval trainings':ab,ti) | Embase/ 13 November 2025 |

**Supplementary Table 3.** Egger's test(HDL)

| Std_Eff | Coefficient | Std. err. | t | P>\|t\| | [95% conf. | interval] |
| --- | --- | --- | --- | --- | --- | --- |
| slope | 57.23359 | 20.29791 | 2.82 | 0.023 | 10.42652 | 104.0407 |
| bias | 162.9733 | 126.9728 | 1.28 | 0.235 | -129.8265 | 455.7731 |

**Supplementary Table 4.** Egger's test(TC)

| Std_Eff | Coefficient | Std. err. | t | P>\|t\| | [95% conf. | interval] |
| --- | --- | --- | --- | --- | --- | --- |
| slope | -1.321586 | .3408334 | -3.88 | 0.004 | -2.092605 | -.5505675 |
| bias | 3.22419 | 1.107621 | 2.91 | 0.017 | .7185765 | 5.729804 |
